# Genomic and Functional Characterization of Environmental Strains of SDS-Degrading *Pseudomonas* spp., Providing a Source of New Sulfatases

**DOI:** 10.3389/fmicb.2018.01795

**Published:** 2018-08-17

**Authors:** Ewa M. Furmanczyk, Leszek Lipinski, Andrzej Dziembowski, Adam Sobczak

**Affiliations:** ^1^Institute of Biochemistry and Biophysics, Polish Academy of Sciences, Warsaw, Poland; ^2^Institute of Genetics and Biotechnology, Faculty of Biology, University of Warsaw, Warsaw, Poland

**Keywords:** comparative genomics, environmental stress response, gene expression, biodegradation, xenobiotics, sodium dodecyl sulfate, alkyl sulfatase, *Pseudomonas* sp.

## Abstract

Biochemical, physiological and genomic comparisons of two *Pseudomonas* strains, assigned previously to the *Pseudomonas jessenii* subgroup, which are efficient SDS-degraders were carried out. A GO enrichment analysis showed that the genomes of SDS-degraders encode more genes connected with bacterial cell wall biosynthesis and alkanesulfonate monooxygenase activity than their closest relatives from the *P. jessenii* subgroup. A transcriptomic analysis of the most promising strain exposed to detergent suggests that although SDS can be later utilized as a carbon source, in early stages it influences cell envelope integrity, causing a global stress response followed by cell wall modification and induction of repair mechanisms. Genomes of the analyzed strains from *P. jessenii* group encode multiple putative sulfatases and their enzymatic activity was experimentally verified, which led to the identification of three novel enzymes exhibiting activity toward SDS. Two of the novel alkylsulfatases showed their highest activity at pH 8.0 and the temperature of 60°C or 70°C. One of the enzymes retained its activity even after 1 h of incubation at 60°C. Ions like K^+^ and Mg^2+^ enhanced enzymatic activity of both proteins, whereas Cu^2+^ or EDTA had inhibitory effects.

## Introduction

Sulfur is one of the essential elements for living organisms. It is required for methionine and cysteine synthesis but is also crucial as a cofactor for many enzymes such as coenzyme A or biotin. Sulfur is common in the environment, but its organic forms are mostly present in sulfate esters or sulfonates which, for instance are not accessible to plants (Kertesz, 2000). Inorganic sulfate is provided mainly by microorganisms possessing enzymes called sulfatases (EC 3.1.6.-), which cleave sulfate ester bonds. This heterogeneous group of enzymes can be divided into at least three distinct classes, depending on the mechanism of catalysis (Kertesz, 2000).

The first group gathers arylsulfatases, proteins with highly conserved consensus motif C/S-X-P-X-A-X_4_-T-G, identified mostly in eukaryotic organisms (Hagelueken et al., 2006). Although, the class name suggests that these enzymes are specific for aromatic substrates, many of them also catalyze desulfurization of sulfated carbohydrates (Toesch et al., 2014). A typical bacterial representative of this group is AtsA from *Pseudomonas aeruginosa* (Boltes et al., 2001). The second group consists of Fe(II) dependent dioxygenases, catalyzing an oxidative cleavage of the sulfate ester to the corresponding aldehyde and inorganic sulfate, with AtsK from *P. putida* as a model example (Müller et al., 2004). The third group includes enzymes with the metallo-β-lactamase-like domain in the N-terminus and SCP-2-like domain in C-terminus. Six sulfatases with different substrate specificities have been characterized to date: SdsA, YraS, and YjcS with activity toward sodium dodecyl sulfate (SDS) (Davison et al., 1992; Liang et al., 2014; Navais et al., 2014), SdsAP and SdsA1 with activity toward primary sulfate esters including sodium dodecyl sulfate (Hagelueken et al., 2006; Long et al., 2011; Schober et al., 2011) and Pisa1 with activity toward secondary alkyl sulfates (Schober et al., 2011). Enzymes from this group differ in the mechanism of action and could cleave either S-O or C-O bond causing the retention or inversion of the product alcohol, which could be verified using chiral or labeled substrates. Early research for SdsA1 and Pisa1 proteins revealed that both sulfatases cleave the C-O bond releasing inverted alcohol. However, they show contrasting substrate specificities for primary (SdsA1) or secondary (PisA1) alkyl sulfates (Schober et al., 2011). Enzymes belonging to the third group of sulfatases are promising candidates for biotechnological tools for SDS decomposition and future industrial applications.

Sodium dodecyl sulfate remains one of the most commonly used anionic detergents and is present in cosmetics, pharmaceuticals, household, and industrial cleaning products, and used in agriculture as an adjuvant to improve spraying properties and pesticide penetration. The extensive application of products containing this surfactant results in its accumulation in various aquatic and terrestrial environments.

The degradation pathway of this detergent was firstly proposed for *Pseudomonas* sp. C12B (Thomas and White, 1989). Experiments using radiolabeled [1-14C] SDS showed that the degradation is initiated by the sulfatase-mediated hydrolysis of sulfur ester to alcohol. Dodecanol is then oxidized to dodecanal, and then further on to dodecanoic acid, which is either elongated to long-chain fatty acids or used as an energy source via the tricarboxylic acid cycle. Further these results were confirmed for another *Pseudomonas* species - *P. aeruginosa* MTCC 10311 using gas chromatography (Ambily and Jisha, 2014). Despite the rapid development of molecular biology techniques till today they have hardly been used to widen our knowledge of SDS degradation and the bacterial cell response toward the detergent. Most of the existing reports are only describing the isolation of bacteria having the capacity to degrade SDS, including a wide variety of *Pseudomonas* strains (Klebensberger et al., 2006; Chaturvedi and Kumar, 2011; Yeldho et al., 2011; John et al., 2015), *Klebsiella oxytoca* (Shukor et al., 2009; Masdor et al., 2015) or microbial consortia (Abboud et al., 2007), derived from various water and terrestrial environments. However, they are mainly focused on the phylogenetic identification of the isolates and the alkylsulfatase activity testing using Native Page Zymography without the detailed enzyme characteristics. Therefore, there are only five well-characterized alkylsulfatases with known sequence and confirmed activity toward SDS (Davison et al., 1992; Hagelueken et al., 2006; Long et al., 2011; Liang et al., 2014; Navais et al., 2014). This year a first attempt to identify the enzymes involved in the further SDS metabolic pathway in *P. aeruginosa* PAO1 was performed. The results from microarray analysis, verified by the characterization of appropriate deletion mutants, led to identification *laoABC* gene cluster coding long-chain alcohols oxidation system responsible for the dodecanol and dodecanal oxidation (Panasia and Philipp, 2018).

In this study we present detailed characterization and comparison of two of five recently identified efficient SDS degrading microorganisms originating from the root zone of a surface flow constructed wetland of a wastewater treatment plant operated by a pesticide packing company in Poland (Furmanczyk et al., 2017c), up to date, the most efficient SDS degrading strains isolated from terrestrial environment. All the isolates assigned to the *P. jessenii* subgroup can be divided into two groups characterized by (1) the number of enzymatic bands visible on Native PAGE Zymography analysis exhibiting SDS desulfurization, and (2) the influence of SDS on growth in culture (Furmanczyk et al., 2017c). The first group formed by strains AP3_10, AP3_20 and AP3_22^T^ (characterized by one band on Native Page Zymography), including the most efficient degrader – AP3_22^T^, were classified as belonging to a novel species – *P. laurylsulfatovorans* (Furmanczyk et al., 2018). The second group contained strains AP3_16 and AP3_19, exhibit two bands on Native Page Zymography.

In this work, we analyzed and compared genomic sequences of two strains AP3_16 and *P. laurylsulfatovorans* AP3_22^T^, differing not only in the resistance to high detergent concentration, but also in the dynamics of SDS degradation. The results of genomic research are presented in the context of their biochemical characteristics and phylogenetic analyzes obtained for the strains AP3_16 and the same set of studies published earlier for the *P. laurylsulfatovorans* AP3_22^T^. These studies allow us to propose the AP3_16 strain as a novel bacterial species called *P. laurylsulfatophila* sp. nov with AP3_16 as a type strain. Further genomic and transcriptomic analyses shed additional light on SDS metabolism and bacterial response mechanisms toward this anionic detergent. Furthermore, genomic analysis of the described microorganisms revealed three novel enzymes with different activities toward SDS. These newly characterized proteins contain an N-terminal metallo-β-lactamase-like domain and a C-terminal SCP-2-like domain, suggesting that they could be classified as alkylsulfatases belonging to the third class of sulfatases. The further characteristics of the sulfatases reported here will be helpful in the development of biochemical tools to manage SDS in either natural or industrial environments.

## Materials and Methods

### Media Preparation

Minimal medium (Furmanczyk et al., 2017c) and lysogeny broth (LB) medium (Sambrook and Russel, 2001) were used. Media were solidified by the addition of 1.5% agar. When necessary, the media were supplemented with kanamycin (50 μg/mL) and/or SDS (1 g/L). The 20% (w/v) SDS and kanamycin (50 mg/mL) stock solutions were prepared in ultrapure water and sterilized by filtration. Unless otherwise indicated, all compounds (used in all media and buffers) were purchased from Sigma-Aldrich, United States.

### Physiological and Biochemical Analyses of the Bacterial Strains

Gram staining was carried out by standard methods. Flagella staining was performed using a wet-mount technique with Ryu stain (Ryu, 1937). Temperature, pH and salinity tolerance of the strains were analyzed by monitoring changes in optical density (OD_600_) in liquid cultures (in comparison to non-inoculated controls). Overnight cultures were diluted in fresh LB media with adjustments for the following assays: pH 7 for temperature assay (4, 8, 15, 23, 30, 37, and 42°C); pH 4.0–10.0 for the pH tolerance analysis or supplemented with NaCl to final concentrations: 0%, 0.5%, 1.0% – 8.0% (with 1% increment) for salinity tolerance analysis. The initial optical density at 600 nm (OD_600_) was 0.05 in each assay. The cultures were incubated with shaking at 145 rpm at 30°C or other tested temperatures (temperature assay). Biochemical features of the microorganisms were determined using a GEN III Biolog microplate (Biolog, United States) and an API 20NE systems (bioMérieux, France) according to manufacturer’s instructions. Degradation of SDS was tested in minimal medium with 0.1% SDS as a sole carbon source and measured by colorimetric assay. Fluorescence pigment production was tested on King B medium (King et al., 1954). Catalase activity was determined by the production of bubbles after addition of 3% (v/v) hydrogen peroxide solution. Oxidase activity was tested using disks containing *N,N*-dimethyl-*p*-phenylenediamine oxolate and α-naphthol (Sigma-Aldrich, United States). As reference strains, *P. laurylsulfatovorans* AP3_22^T^, *P. jessenii* DSM 17150^T^, *P. vancouverensis* DSM 17555^T^ and *P. umsongensis* DSM 16611^T^ were used.

### Chemotaxonomic Analysis

The fatty acid profiles of AP3_16^T^ and other closely related type strains were analyzed at Leibniz Institute DSMZ using the Sherlock Microbial Identification System (MIDI, Microbial ID, United States). Peaks were automatically integrated and fatty acid names and percentages were calculated with the MIS Standard Software (Microbial ID, Sherlock version 6.1, database TSBA40 4.10). All strains were simultaneously grown at 28°C for 2 days on trypticase soy agar (TSA) prior to analysis.

### Colorimetric Assay for SDS Degradation

The SDS concentration was determined in triplicate in 96-well plates using Stains-All reagent as described elsewhere (Rusconi et al., 2001; Furmanczyk et al., 2017c). Samples from each time point were diluted 10 times, and 8 μL of the diluted samples were dispensed into a well, supplemented with 100 μL of water and 100 μL of the Stains-All working solution. Finally, the absorbance (at 438 nm) was measured after 10 min of incubation at RT.

### Genomic DNA Isolation, Library Preparation and Sequencing

For whole genome sequencing of AP3_16^T^ strain, DNA was isolated following a previously described protocol (Furmanczyk et al., 2017a). The isolated genomic DNA was used to prepare two types of libraries: (1) paired-end library with average insert size 500 bp (using KAPA HTP Library Preparation Kit for Illumina platforms according to manufacturer’s protocol) (Kapa Biosystems, United States), (2) Nextera^®^ Mate Pair library with average insert size 8 kbp (using Illumina protocol) (Illumina, United States). The libraries were verified using a 2100 Bioanalyzer (Agilent, United States) High-Sensitivity DNA Assay and KAPA Library Quantification Kit for the Illumina (Kapa Biosystems, United States). Sequencing was performed using an Illumina MiSeq (MiSeq Reagent Kit v3, 600 cycles) (Illumina, United States) with read length of 300 bp.

### Genome Assembly and Annotation

Reads from the sequencing of AP3_16^T^ strain were processed as previously described (Furmanczyk et al., 2017a) – assembled using SPAdes 3.9.0 (Bankevich et al., 2012). Contigs longer than 1 kbp were deposited in Genbank (accession number: NIRS00000000.1) and annotated using NCBI PGAP (Tatusova et al., 2016).

### Phylogenetic Analyses

The phylogenetic analysis was based on the longest common fragment of the 16S rRNA gene sequences (1349 bp) selected from ClustalW alignment of the AP3_16 strain and the 29 closest type strains of other *Pseudomonas* species with sequences obtained from GenBank (accession numbers in **Supplementary Table S1**). Phylogenetic analysis was performed using the MEGA software version X (Kumar et al., 2018), the maximum-likelihood method with 1,000 bootstrap replicates under the Hasegawa-Kishino-Yano model (Hasegawa et al., 1985) using a discrete Gamma distribution (+G) and assuming a fraction of sites are invariable (+I). All positions containing gaps or missing data were removed, what resulted in a 1297 bp sequence in the final dataset.

The multilocus sequence analysis (MLSA) of the sequence fragments of three housekeeping genes (*gyrB, rpoD*, and *rpoB*) together with the 16S rRNA gene sequence was done for detailed AP3_16^T^ strain identification as described elsewhere (Furmanczyk et al., 2018). Sequences of type strains from the database were retrieved using BLAST (accession numbers in **Supplementary Table S1**). Partial sequences of the *gyrB, rpoD*, and *rpoB* genes of the SDS degrading strain were retrieved from the sequenced genome. Phylogenetic analysis was performed using the MEGA software version X, the maximum-likelihood method with 1,000 bootstrap replicates under the General Time Reversible model (Nei and Kumar, 2000) using a discrete Gamma distribution (+G) and assuming a fraction of sites are invariable (+I).

### Comparative Genomics

The relatedness of the *P. laurylsulfatophila* AP3_16^T^ genome to other public genomes of closely related *Pseudomonas* species was determined based on the Average Nucleotide Identity using BLASTn (ANIb) using the JSpecies software (Richter et al., 2016) and Genome-to-Genome-Distance (GGDC 2.0), which was calculated using the web service (Meier-Kolthoff et al., 2013) with the recommended BLAST+ method. The draft genome sequence of *P. laurylsulfatophila* AP3_16^T^ (GenBank accession no. NIRS00000000.1) was compared to the draft genomes of *P. laurylsulfatovorans* AP3_22^T^ (GenBank: MUJK00000000.1), *P. jessenii* DSM 17150^T^ (Genbank: NIWT00000000.1), *P. umsongensis* DSM 16611^T^ (Genbank: NIWU00000000.1) (Furmanczyk et al., 2017b) and *P. vancouverensis* DSM 17555^T^ (IMG Taxon ID: IMG 2667527228). The OrthoVenn web server (Wang et al., 2015) was used to identify orthologous clusters among the genomes of the five strains. Additionally, the COGs (Clusters of Orthologous Groups) were assigned to functional categories for each single genome using COGNIZER software (Bose et al., 2015).

### RNA Manipulation and the Transcriptional Deep Sequencing Data Analysis

#### Bacterial Growth and SDS Degradation Assay for Transcriptomic Analysis

For the transcriptomic analysis, the *P. laurylsulfatovorans* AP3_22^T^ strain was precultured overnight in LB medium. Then it was washed with minimal medium and diluted in the fresh 0.1 LB medium with or without SDS (5 g/L) to the initial OD_600_ = 0.4 and cultured in 30°C with 140 rpm agitation. Every 30 min the cultures OD_600_ and SDS concentration (see section “Colorimetric Assay for SDS Degradation”) were measured and the samples for RNA isolation were collected. The experiment lasted for 150 min. All the measurements and sample collection were made in triplicate.

#### RNA Isolation

Pellets from 5 ml of culture from each time point were resuspended in 1 ml of TE buffer and transferred to a new tube with 250 μl of zirconia beads. One ml of TRIzol was added and each sample was mixed by vortexing for 3 min followed by adding 200 μl of chloroform and short mixing (30 s vortex). After 30 min of incubation at RT, the samples were centrifuged (10 min, 14000 rpm, 4°C). RNA was precipitated with isopropanol and resuspended in 40 μl of water. The concentration and purity of RNA solutions were determined by NanoDrop (Thermo Fisher Scientific, United States). The RNA quality was evaluated by agarose gel electrophoresis. Ten μg of RNA was treated with TURBO DNase (Thermo Fisher Scientific, United States) according to the manufacturer’s protocol followed by phenol/chloroform extraction. The purified samples were stored at -80°C.

### RNA Libraries Preparation, Sequencing and Bioinformatic Analysis

RNA libraries were prepared using a KAPA Stranded RNA-Seq Library Preparation Kit (Kapa Biosystems, United States). Two and a half μg of total RNA was used as input. rRNA was removed using Ribo-Zero rRNA Removal Kit (Bacteria) (Illumina, United States). Fragmentation and priming were done at 94°C for 8 min. The libraries were verified using a 2100 Bioanalyzer (Agilent, United States) High-Sensitivity DNA Assay and KAPA Library Quantification Kit for the Illumina (Kapa Biosystems, United States). Sequencing was performed using an Illumina NextSeq (2 × 75 bp) (Illumina, United States) resulting an average of 5 M of paired reads per library. Reads were filtered to quality score (Q30) and length (>40 bp), and the adaptor sequences were removed using the Cutadapt script (Martin, 2011). The 16S rRNA reads were filtered out using SortMeRNA (Kopylova et al., 2012) and the remaining sequences were mapped to the reference draft genome of *P. laurylsulfatovorans* AP3_22^T^ (Accession number: MUJK00000000) using Bowtie2 (Langmead and Salzberg, 2012). Then reads mapping to the CDS regions were counted using the htseq-count script (Anders et al., 2015). Differential expression analyses were performed using the DESeq2 Bioconductor R package (Love et al., 2014). Associated data have been deposited to the NCBI Gene Expression Omnibus and are available under GSE110573 accession number.

### Cloning Sulfatase Genes With Potential Promoter Regions and Functionality Verification in *Escherichia coli*

DNA sequences coding potential sulfatases with putative promoter regions were amplified using designed primer pairs (**Supplementary Table S2**). The genomic DNA of *P. laurylsulfatophila* AP3_16^T^ or *P. laurylsulfatovorans* AP3_22^T^ were used as the PCR templates. The PCR (50 μL) was performed in a TProfessional Basic Thermocycler (Analytik Jena AG, Germany), using 0.02 U/μl Phusion High-Fidelity DNA Polymerase (Thermo Fisher Scientific, United States) supplied with 1x HF-buffer, 0.2 mM dNTP mixture and primers (0.2 μM each). PCR reaction conditions were: one cycle at 99°C for 5 min; 35 cycles at 99°C for 30 s, 56°C for 30 s, and 72°C for 30–90 s (details in **Supplementary Table S2**) with final extension at 72°C for 5 min. The PCR products were purified using AMPureXP (Beckman Coulter, United States) with a 0.8:1 ratio, then cloned into pCR^TM^Blunt II-TOPO^®^ (Invitrogen, United States) and transformed into *E. coli* TOP10. The constructs were analyzed by digestion with EcoRI restriction enzyme (Fast Digest enzyme; Thermo Fisher Scientific, United States) according to the manufacturer protocol. Next, Sanger sequencing of the inserts was done. The overnight LB-precultures of *E. coli* TOP10 strains carrying the cloned sulfatases were used to inoculate fresh LB medium (1:50 v/v) with kanamycin and SDS (1 g/L). After 24 h of incubation, the SDS concentration in the media was determined (see section “Colorimetric Assay for SDS Degradation”). The measurements were made in triplicates.

### Expression of Recombinant Proteins and SDS Degradation Assay

The four selected genes (CD175_04445, CD175_09595, CD175_30230 and B0D71_15760) were amplified with or without the predicted signal sequence using designed primers pairs (**Supplementary Table S2**). PCR (50 μL) was performed using Phusion High-Fidelity DNA Polymerase (Thermo Fisher Scientific, United States; supplied with 1× HF-buffer and other components as previously). The PCR involved: one cycle at 99°C for 5 min; 10 cycles at 99°C for 30 s, 56°C for 30 s, and 72°C for 65 s; 20 cycles at 99°C for 30 s, 64°C for 30 s, and 72°C for 65 s; with final extension at 72°C for 5 min. PCR products were purified using AMPureXP (Beckman Coulter, United States) with a 0.8:1 ratio, then cloned into pET28 derived vector, which adds a TEV protease cleavable C-terminal HisTag, using a sequence- and ligation-independent cloning protocol (Jeong et al., 2012). The plasmids were analyzed by digestion with restriction enzymes (NcoI and XhoI, both Fast Digest enzymes; Thermo Fisher Scientific, United States) according to manufacturer protocol. Next Sanger sequencing of the inserts was done. Positively verified vectors were transferred into *E. coli* BL21 (RIL) for overexpression. The overnight LB-precultures of *E. coli* BL21 (RIL) strains carrying vectors with cloned sulfatases were used to inoculate fresh LB medium (1:50 v/v) with kanamycin. After 2 h of incubation at 37°C, the cultures were cooled to 18°C and isopropyl β-D-1-thiogalactopyranoside (IPTG) to final concentration of 0.5 mM was added. After 2 h of incubation at 18°C, SDS to final concentration of 0.1% was added. After 18 h of additional incubation the SDS concentration in the media was determined (see section “Colorimetric Assay for SDS Degradation”). The measurements were carried out in triplicates.

### Bioinformatic Analyses of the Alkylsulfatases Sequences

The alignment of protein sequences was prepared using Muscle (Edgar, 2004) and the phylogenetic analysis was performed using the MEGA X version, the maximum-likelihood method with 1,000 bootstrap replicates under the Le_Gascuel_2008 (Le and Gascuel, 2008) using a discrete Gamma distribution (+G). The signal peptide in the deduced amino acid sequences was predicted using SignalP 4.1 Server^1^ (Nielsen, 2017). The estimated molecular weight and pI of the proteins were calculated using Compute pI/Mw ExPASy server tool (Gasteiger et al., 2005).

### Purification of Recombinant Sulfatases

The overnight LB-precultures of *E. coli* BL21 (RIL) strains carrying the cloned sulfatases were used to inoculate LB medium (1:50 v/v) with kanamycin. After 2 h of incubation at 37°C, the cultures were cooled and IPTG to final concentration of 0.5 mM was added. After 20 h of incubation at 18°C, the cells were harvested by centrifugation. Cell pellets were resuspended in lysis buffer (50 mM HEPES, 300 mM NaCl, 20 mM imidazole, 50 μM PMSF, 10 mM β-mercaptoethanol, 0.1% Tween 20, 10% glycerol and 50 μg/mL lysozyme, pH 7.5). Cells were lysed via sonication in the Bioruptor Plus sonication system (Diagenode, Belgium) in a cooled water bath (4°C) at high power (300 W) for 30 cycles of 30 s on and 30 s off. Debris was removed by centrifugation and the supernatant was filtered through 0.22 μm membrane. The protein was purified using Protino 96 Ni-IDA (Macherey-Nagel, Germany). Clarified lysate was loaded onto preactivated column (Protino 96 Ni-IDA). The resin was washed with 50 column volumes of wash buffer I (50 mM HEPES pH 7.5, 300 mM NaCl, 10 mM MgCl_2_) and then with 25 column volumes of wash buffer II (50 mM HEPES pH 7.5, 300 mM NaCl). Protein was eluted with three fractions (300 μl each) of elution buffer (50 mM HEPES, 300 mM NaCl, 10% glycerol, 250 mM imidazole pH 7.5). Fractions containing the purified protein were dialyzed against dialysis buffer (50 mM HEPES, 300 mM NaCl, 10% glycerol, 10 mM β-mercaptoethanol, pH 7.5). Protein concentration was measured using Bio-Rad Protein Colorimetric Assay (Bio-Rad, United States) with BSA as a standard.

### Enzyme Activity Assay

The activity of each alkylsulfatase was assayed using Stains-all reagent as mentioned earlier. Two μL of enzyme solution (150 μg/mL) was mixed with 100 μL of 50 mM Tris-HCl buffer containing SDS with a final concentration of 0.01% (w/v). After incubation at 37°C for 5 min, the reaction was terminated by incubation of the sample at 100°C for 5 min. The SDS concentration was determined in a 96-well plate using Stains-All reagent as described previously (Furmanczyk et al., 2017c), except the dilution step was omitted.

### Effects of pH and Temperature on Sulfatases Activity

The optimal temperature for the activity of the purified sulfatases was determined by carrying out the enzyme activity assay in the range of temperatures (4–90°C) in 50 mM Tris-HCl buffer (pH 8.0) for 5 min. The optimal pH of sulfatases was assayed at 37°C in a pH range of 4.0 – 10.0 (with citrate buffer for pH 4–5; citrate-phosphate buffer for pH 6; Tris-HCl buffer for pH 7–8; glycine-NaOH buffer for pH 9–10). The relative activity was defined as the percentage of activity determined with respect to the maximum activity of each sulfatase. All of the measurements were made in triplicate.

### Effects of Temperature on Sulfatase Stability

The thermostability of the recombinant enzymes was determined by measuring in triplicates the residual activity of the enzyme, exposed to five different temperatures: 40, 50, 60, and 70°C for 1 h and 100°C for 10 min. After the incubation time, the remaining activity was assayed under standard conditions.

### Effects of Various Reagents on Sulfatases Activity

The effect of ions: Ca^2+^, Cu^2+^, Mg^2+^, Mn^2+^, Na^+^, K^+^ used as chloride salts, and EDTA on the sulfatases activity was tested. The specified concentrations (10 mM and/or 100 mM) of the mentioned reagents were added to the reaction mixture and the enzyme’s activity was measured at 37°C and pH 8.0. In each case, the relative activity was defined as the percentage of activity determined in the standard conditions without any additive.

## Results

### Genome Sequencing and Phylogenetic Analyses

Based on a phylogenetic analysis of 16S rRNA sequences, strains studied in this work were previously assigned to the *P. jessenii* subgroup (Furmanczyk et al., 2017b) and the subsequent characterization of strain AP3_22^T^ was based on its genome sequence, allowing for description as a new species (Furmanczyk et al., 2018). As part of this work, the AP3_16 genome has also been sequenced to identify genes involved in response towards SDS and improve the strain classification.

Sequencing was performed on the Illumina MiSeq platform, with 300 bp read-length, resulting in an assembly with estimated average genome coverage of 103×. The draft genome of the AP3_16 strain consists of 13 contigs, containing 6,684,644 bp with a GC content of 60.1% and is 88.65% coding. The genome encodes 6074 predicted genes from which 5879 (96.79%) are protein coding sequences and 80.18% have a putative assigned function, while the remaining 19.82% encode hypothetical proteins. The draft genome of the AP3_16 strain has 70 RNA genes: 8 rRNAs, 58 tRNAs, 4 ncRNAs, and 125 pseudogenes. No plasmid sequences were detected.

**FIGURE 1:**
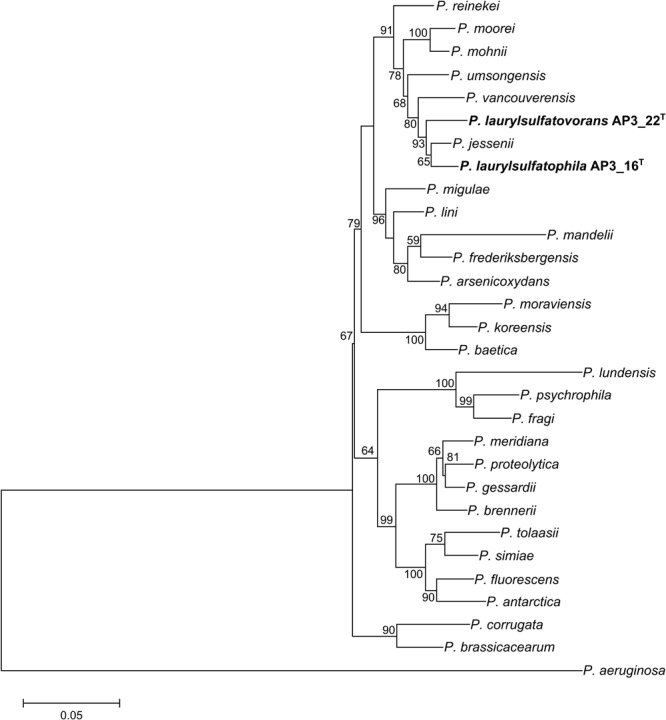
Phylogenetic tree representing the relative position of two SDS degrading strains. Phylogenetic tree constructed using concatenated partial sequences of 16S rRNA, *gyrB, rpoD*, and *rpoB* genes representing the relative position of *Pseudomonas laurylsulfatophila* AP3_16^T^ and *P. laurylsulfatovorans* AP3_22^T^ strains (in bold) in the *Pseudomonas* genus. All positions containing gaps or missing data were eliminated. The final dataset contained 3,563 positions. Accession numbers of sequences used in this study are listed in the **Supplementary Table S1**. Bootstrap values are represented at the branching points (only values above 50% are shown). The bar represents 0.05 substitutions per site.

Phylogenetic analysis of the AP3_16 strain based on the combined multilocus sequence analysis (MLSA): partial sequences of housekeeping genes (*gyrB, rpoD*, and *rpoB*) and 16S rRNA showed that the isolate shares the highest similarity (97.69%) with *P. jessenii* DSM 17150^T^. The next closest type strains were: *P. laurylsulfatovorans* AP3_22^T^ (96.83%), *P. vancouverensis* DSM 17555^T^ (96.5%), and *P. umsongensis* DSM 16611^T^ (96.47%), which all belong to the *P. jessenii* subgroup. The phylogenetic analysis based on MLSA showed that AP3_16 strain clusters with a group within the *P. jessenii* subgroup (**Figure 1**). However, other genomic comparisons (ANIb and GGDC), routinely performed during novel bacterial species classification, yielded the highest percentage similarity with *P. laurylsulfatovorans* AP3_22^T^ (91.15% for ANIb and 46% for dDDH), similarly to the phylogenetic analysis based only on the 16S rRNA gene fragment (**Supplementary Figure S1**). Nonetheless, both values suggest that the AP3_16 isolate should be regarded as separate species (Chun et al., 2018) for which we propose the name *P. laurylsulfatophila* sp. nov. with AP3_16^T^ as a type strain.

### Physiological and Biochemical Analyses of the Bacterial Strains

One of the main goal of this study was to investigate and compare the metabolic potentials for SDS degradation encoded in the genomes of the two strains. However, genomic characterization should only be considered as a promising but unconfirmed potential. Therefore, we analyzed biochemical properties of the characterized isolates AP3_16^T^ and *P. laurylsulfatovorans* AP3_22^T^ and compared them to the genomic information. The biochemical analyses included the Biolog GEN III Microplate panel, the API 20 NE system, production of fluorescence pigments on King B medium, the ability to degrade SDS, and testing for catalase and oxidase activities. To gain additional information from this comparison we included the three closest relatives within the *P. jessenii* subgroup: *P. jessenii* DSM 17150^T^, *P. vancouverensis* DSM 17555^T^ and *P. umsongensis* DSM 16611^T^. The results were summarized in **Table 1** and **Supplementary Table S3**. Also, the fatty acid profiles of AP3_16^T^ and other closest type strains were compared (**Supplementary Table S4**).

**Table 1:** Physiological and biochemical characteristics of *Pseudomonas laurylsulfatophila* AP3_16^T^ that differentiate this strain from the other closest *Pseudomonas* type strains.

Carbon source	1	2	3	4	5
Dextrin	+	–	–	–	–
Sucrose	+	+	–	–	–
*N*-acetyl-D-glucosamine	+	+	+	–	+
D-galactose	+	+	+	+	–
D-fucose	+	–	–	–	+
L-fucose	+	–	–	–	+
Inosine	–	–	–	–	+
D-mannitol	+	–	+	–	+
D-arabitol	+	–	+	–	+
D-fructose-6-PO_4_	+	–	–	–	–
D-aspartic acid	–	–	+	–	–
D-serine	+	+	+	–	+
Glycyl-L-proline	–	–	+	+	–
L-pyroglutamic acid	+	+	+	+	–
Pectin	+	+	+	+	–
D-galacturonic acid	+	+	–	+	–
L-galacturonic acid lactone	+	+	–	+	–
D-glucuronic acid	+	+	–	+	–
Glucuronamide	+	+	–	+	+
Quinic acid	+	+	+	+	–
D-Saccharic Acid	+	+	+	+	–
*p*-hydroxy-phenylacetic acid	–	–	+	–	–
Tween 40	+	+	+	+	–
α -keto-butyric acid	+	+	–	–	–
Acetoacetic acid	+	+	–	–	–
**Other biolog Gen III tests**	**1**	**2**	**3**	**4**	**5**
4% NaCl	+	+	–	+	+
8% NaCl	–	–	–	+	–
Sodium butyrate	+	–	–	–	–
Sodium bromate	+	+	–	+	+
**API20 NE feature**	**1**	**2**	**3**	**4**	**5**
Nitrate reduction	–	+	+	+	+
Arginine dehydrolase	–	–	–	+	–
Arabinose	–	+	+	+	–
Mannitol	+	–	+	–	+
*N*-acetyl-glucosamine	+	+	+	–	+
**Other tests**	**1**	**2**	**3**	**4**	**5**
SDS degradation	+	+	–	–	+

*Lanes: 1, P. laurylsulfatophila AP3_16^*T*^; 2, P. laurylsulfatovorans AP3_22^*T*^; 3, P. jessenii CIP 105724^*T*^; 4, P. umsongensis DSM 16611^*T*^; 5, *P. vancouverensis* DSM 17555^*T*^. +, positive; -, negative. The strains were tested using Biolog GEN III Microplate, API20 NE strips and other tests characterized in the Materials and Methods section*.

**FIGURE 2:**
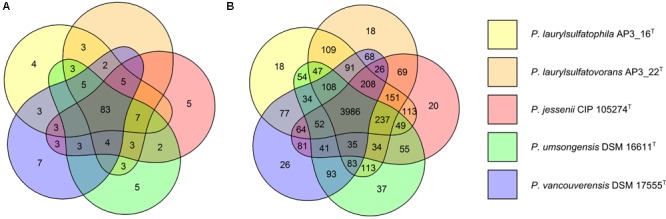
Comparison of biochemical, physiological and genomic potential of the five *Pseudomonas* strains. Venn diagrams based on: **(A)** the results of the physiological and biochemical tests for the five analyzed strains, the number of main features differentiating the strains are presented on the diagram; **(B)** the number of identified orthologous clusters between the genomes of the five compared strains. The numbers indicate the identified unique or shared orthologs between the strains.

The five strains taken for the analysis share 83 of the 118 tested features (**Figure 2A**) including, typical for *Pseudomonas*, the ability to utilize simple sugars such as glucose, mannose and fructose and utilize amino acids and short chain carboxylic acids (including acetic acid, propionic acid, L-malic acid, citric acid or L-lactic acid). All strains also share multidrug resistance to vancomycin, lincomycin, troleandomycin, rifamycin Sv, fusidic acid, nalidixic acid and aztreonam. The five strains yielded positive results in more than half of the conducted tests (67 for *P. vancouverensis* DSM 17555^T^, 70 for *P. jessenii* DSM 17150^T^ and *P. umsongensis* DSM 16611^T^, 74 for *P. laurylsulfatovorans* AP3_22^T^ and 80 for the AP3_16^T^ strain). However, all isolates were unable to decompose a similar pattern of substrates including many disaccharides (for example: D-maltose, D-trehalose, D-cellobiose, gentiobiose, D-turanose, D-melibiose), D-sorbitol, gelatin or lack specific enzymatic activities like urease, β-galactosidase. None of the microorganism was observed to ferment glucose, produce indole or hydrolyze esculin or gelatin.

The AP3_16^T^ strain could be easily differentiated from the other four strains by its ability to use dextrin or D-fructose-6-PO_4_ as the sole carbon source, growth in the presence of sodium butyrate and the inability to reduce nitrates, which strongly supports its classification as a novel species. *P. jessenii* DSM 17150^T^ was the only strain capable of assimilating *p*-hydroxy-phenylacetic acid and D-aspartic acid but was not able to assimilate glucuronamide and did not grow at or above 4% NaCl salinity, or in the presence of sodium bromate. *P. umsongensis* DSM 16611^T^ was the only strain that could not use D-serine or *N*-acetyl-D-glucosamine as a sole carbon source. However, it was the only strain able to grow in the presence of 8% NaCl and showed arginine dehydrolase activity. *P. vancouverensis* DSM 17555^T^ was the only strain able to assimilate inosine but was unable to use: D-galactose, L-pyroglutamic acid, pectin, quinic acid, D-saccharic acid or Tween20 as a sole carbon source. Only one strain - *P. laurylsulfatovorans* AP3_22^T^, could not be easily distinguished from the other tested microorganisms. However, it shared many features with another SDS decomposing strain, AP3_16^T^, such as the assimilation of D-sucrose and α-keto-butyric acid, and the ability to use acetoacetic acid as a carbon source. These features separated these strains from the others. Interestingly, in addition to *P. laurylsulfatovorans* AP3_22^T^ and AP3_16^T^, *P. vancouverensis* DSM 17555^T^ was also found to decompose SDS.

### Genomic Comparison of the Bacterial Strains

We performed a comprehensive comparison of the draft genome sequences of AP3_16^T^ and AP3_22^T^ strains with three other type strains (summarized in **Table 2**), which revealed that all of the microorganisms belonging to the *P. jessenii* subgroup are characterized by a 6.4–6.7 Mbp genome with similar GC contents (59.6–60.1%). Also, the total number of predicted genes was similar, ranging from 5929 to 6152 genes. Assignment of genes into COG (Clusters of Orthologous Groups) functional categories did not show significant differences among the strains (**Supplementary Table S5**). We used the OrthoVenn web server with the Gene Ontology (GO) enrichment analysis to identify the overlap among the orthologous clusters of predicted protein sequences within the five strains.

**Table 2:** General statistics and the relatedness of the five compared genomes.

Attribute	1	2	3	4	5
Genome Size	6 684 644	6 686 052	6 537 206	6 701 403	6 424 905
GC [%]	60.1	59.6	59.7	59.7	60.1
Contigs	13	30	13	14	1
ANIb^∗^		91.15	90.97	84.99	86.06
dDDH^∗∗^		46.00	45.8	32.0	32.90
Total genes	6074	6118	6019	6152	5929
Protein coding genes	5879	5877	5826	5930	5835
RNA genes	70	77	72	73	94
Pseudo genes	125	164	121	150	74
Genes assigned to COGs	4791	4794	4710	4791	4730

*Lanes: 1, P. laurylsulfatophila AP3_16^*T*^; 2, P. laurylsulfatovorans AP3_22^*T*^; 3, P. jessenii CIP 105724^*T*^; 4, P. umsongensis DSM 16611^*T*^; 5, P. vancouverensis DSM 17555^*T*^. ^∗^ANIb threshold for species definition: ≥95%; values calculated with P. laurylsulfatophila AP3_16^*T*^ as a reference. ^∗∗^GGDC threshold for species definition: ≥70%; values calculated with P. laurylsulfatophila AP3_16^*T*^ as a reference.*

From 6078 orthologous clusters formed based on the five strains comparison, the Venn diagram (**Figure 2B**) shows that 3986 gene clusters are shared by all five strains, which corresponds to 73.8–78.8% of all identified clusters for each strain. The diagram also shows that *P. laurylsulfatophila* AP3_16^T^ shares more orthologous gene clusters with *P. laurylsulfatovorans* AP3_22^T^ (4937 hits) than with *P. jessenii* DSM17150^T^ (4860 hits) or the other strains included in the analysis (4620 or 4567 hits for *P. vancouverensis* DSM 17555^T^ or *P. umsongensis* DSM 16611^T^, respectively). Although *P. laurylsulfatophila* AP3_16^T^ and *P. jessenii* DSM17150^T^ share 113 unique gene clusters, these features are not directly related to any specific catabolic processes, but rather to various transporters involved in ion or drug transport. *P. laurylsulfatophila* AP3_16^T^ and *P. laurylsulfatovorans* AP3_22^T^ strains share 109 specific orthologous clusters not present in any other analyzed strain. GO analysis of these clusters showed that some of the significantly enriched genes could be involved in sucrose metabolism or possess alkanesulfonate monooxygenase activity. On the other hand, *P. laurylsulfatovorans* AP3_22^T^ did not show significant enrichment of genes with assigned molecular function or any biological process. However, analysis of the gene clusters strongly suggests that the three strains: *P. laurylsulfatophila* AP3_16^T^, *P. laurylsulfatovorans* AP3_22^T^, and *P. jessenii* DSM17150^T^ are the closest relatives from among all the microorganisms studied here. They share 4582 clusters, 151 of which are uniquely present in those three isolates.

The GO analysis performed for all strains with identified SDS-degrading features (*P. laurylsulfatophila* AP3_16^T^, *P. laurylsulfatovorans* AP3_22^T^, and *P. vancouverensis* DSM 17555^T^) showed enrichment of clusters associated with lipopolysaccharide and lipid A biosynthesis, lipid glycosylation (a set of genes related to biosynthesis of bacterial cell wall components) or other clusters with potential alkanesulfonate monooxygenase activities. However, there are some genes linked to arylsulfatase activity characteristic only for *P. laurylsulfatophila* AP3_16^T^ and *P. vancouverensis* DSM 17555^T^. Intriguingly, another cluster with assigned alkanesulfonate monooxygenase activity was also enriched in *P. laurylsulfatophila* AP3_16^T^, *P. vancouverensis* DSM 17555^T^ and *P. umsongensis* DSM 16611^T^ (the strain without SDS degrading ability).

There are other findings that support the physiological and biochemical characterization of the five strains. For example, the ability of nitrate reduction by *P. laurylsulfatovorans* AP3_22^T^, *P. jessenii* DSM17150^T^, *P. umsongensis* DSM 16611^T^ and *P. vancouverensis* DSM 17555^T^ was in accordance with the enrichment of gene clusters associated with those features.

### Transcriptomic Analysis

Previous results (Furmanczyk et al., 2017c) suggest that in the presence of SDS detergent degradation activity can be induced in the AP3_16 strain, while induction in the *P. laurylsulfatovorans* AP3_22^T^ does not depend on the presence of SDS in the medium. We wanted to confirm whether the expression of potential alkylsulfatases was uncorrelated with the presence of SDS. Moreover, for the AP3_22^T^ strain we wanted to determine other genes involved in the bacterial cell response to SDS presence in the environment.

To investigate the global response and the physiological consequences of the SDS treatment, the *P. laurylsulfatovorans* AP3_22^T^ strain was cultured in the presence of detergent, followed by RNA sequencing and differential gene expression analysis. According to the degradation assay and the OD_600_ measurements, the SDS did not affect the growth of the AP3_22^T^ strain (**Supplementary Figure S2**). The significant detergent degradation began 30 min after the initiation of the experiment and lasted for the next 120 min, after which, the SDS was almost fully degraded. Therefore, for the detailed differential expression analysis the following time points were selected: T5 (5 min – fast response to SDS), T30 (still no degradation observed), T60 (in the middle of degradation process) and T150 (the end of the experiment – detergent almost fully degraded).

Data from the RNA sequencing were filtered to quality scores and mapped back to the annotated draft DNA genome sequence of the *P. laurylsulfatovorans* AP3_22^T^ (GenBank: MUJK00000000.1) as the reference and further analyzed using DESeq2 package in R. A criterion of significant differential expression |log_2_ fold change| > 1 and *p*_adj_ < 0.0001 was chosen. Significant changes in gene expression were observed at each time point (**Supplementary Table S6**).

Bacterial response to detergent was apparent even at the first time-point (T5), demonstrated by 14 upregulated and 3 downregulated genes in comparison to the control culture without SDS (**Supplementary Table S6**). At this first stage, upregulated genes were associated with the stress responses like (a) polyphosphate kinase 2 (B0D71_15235) involved in polyphosphates synthesis, which could act as signal molecules during starvation or oxidative and osmotic stresses; (b) cardiolipin synthase (B0D71_17945); (c) glycerol-3-phosphate dehydrogenase (BD071_19845); (d) amidase (B0D71_26625) involved in the synthesis of cell membranes components (like phospholipids). Interestingly, the gene coding acyl-CoA desaturase (B0D71_22785), involved in unsaturated fatty acid synthesis, was downregulated.

At the second time point (30 min of SDS treatment), before detergent degradation had been observed, 13 genes were upregulated and 14 were downregulated (**Supplementary Table S6**). The highest positively regulated genes products are the most probably involved in the stress response, like: B0D71_26505 (NUDIX hydrolase), B0D71_01775 (lactoylglutathione lyase) or in cell wall metabolism (B0D71_21570 – anhydro-*N*-acetylmuramic acid kinase). Moreover, a gene (B0D71_25530) associated with polyhydroxyalkanoate (PHA) production is also positively regulated. PHAs are storage compounds of carbon and energy in bacteria. This suggests that after 30 min in the presence of SDS PHA biosynthesis was initiated. Upregulation of a key enzyme involved in promoting phospholipid synthesis (B0D71_06340 – phosphatidic acid phosphatase) was also observed. On the other hand, the downregulated genes were those involved in fatty acid catabolism like branched-chain alpha-keto acid dehydrogenase subunit E2 (B0D71_10300) or 2-methylisocitrate lyase (B0D71_08420) required for the utilization of propionyl-CoA (the metabolite of long fatty acids degradation).

After 60 min, when SDS degradation was underway, 23 genes were upregulated and 13 were downregulated (**Supplementary Table S6**). At this time point, the B0D71_25530 gene related to PHA accumulation was still upregulated and a rapid shift in the expression of global metabolic categories took place. This time the previously upregulated genes involved in stress response (B0D71_26505), phospholipids synthesis (B0D71_19845) and lipid synthesis (B0D71_24405 or B0D71_19855) were downregulated. Conversely, the genes potentially involved in fatty acids catabolism (B0D71_10300 or B0D71_21630) and other forms of stress response, like chaperones involved in the protein folding (B0D71_20535) were upregulated.

At the end of the experiment when the SDS was completely degraded, 37 genes were upregulated and 44 were downregulated, resulting in redirection of the global metabolism from fatty acids utilization (for details see **Supplementary Table S6**) to lipopolysaccharide biosynthesis (B0D71_23925). Also upregulated were genes connected to amino acids metabolism (B0D71_08225, B0D71_11870, B0D71_08240 – coding the amino acid transporters, B0D71_12805 or B0D71_18785 coding L-asparaginase or lysine methyltransferase, respectively) and diverse stress response (B0D71_20005, B0D71_112865, B0D71_27080, B0D71_06515). Many of these activities are critically involved with the process entering the stationary growth phase. However, according to optical density, the detergent did not affect the growth of AP3_22^T^ – there was no significant difference in cell density growth between the SDS degrading culture and the control culture, so both of studied variants were probably at the same growth phase, which makes the results from the differential gene expression analysis ambiguous. Expression of any genes assigned as sulfatases did not significantly change at any time point.

### Insights From the Genomes of SDS Degrading Strains – Identification of Enzymes Involved in SDS Desulfurization

Among the characterized strains (AP3_16^T^ and AP3_22^T^), 1-dodecanol is the main by-product formed during SDS degradation, which suggests that alkylsulfatases were responsible for the first step of SDS metabolism (Furmanczyk et al., 2017c).

According to the NCBI annotation in the genome of *P. laurylsulfatophila* AP3_16^T^, we found 15 complete sequences coding putative sulfatases and one partial sequence of such an enzyme, while the genome of *P. laurylsulfatovorans* AP3_22^T^, harbored only six such sequences. A BLASTP search revealed that the sulfatases encoded in the genomes of the two SDS degrading strains represent two of the three classes of bacterial sulfatases. Most of the putative enzymes (17 proteins) share the arylsulfatase A domain – a typical region for the first group of sulfatases. Four proteins: three from AP3_16 strain (CD175_04445, CD175_30230, and CD175_09595) and one from AP3_22^T^ strain (B0D71_15760) belong to the alkylsulfatases – the third group of sulfatases with confirmed activity toward primary or secondary alkyl sulfates. We attempted to verify which of the enzymes similar to alkylsulfatases are responsible for SDS desulfurylation in the strains. Expressing plasmids containing complete gene sequences of the potential sulfatases including putative, native promoter regions were transformed into the *E. coli* TOP10 and the degrading capabilities of these strains were tested. Only two strains, carrying the sulfatases belonging to the third group of bacterial sulfatases (CD175_09595 and B0D71_15760), could decompose SDS (**Supplementary Figure S3**). Therefore, in further experiments we focused only on the proteins from the third group of sulfatases.

**FIGURE 3:**
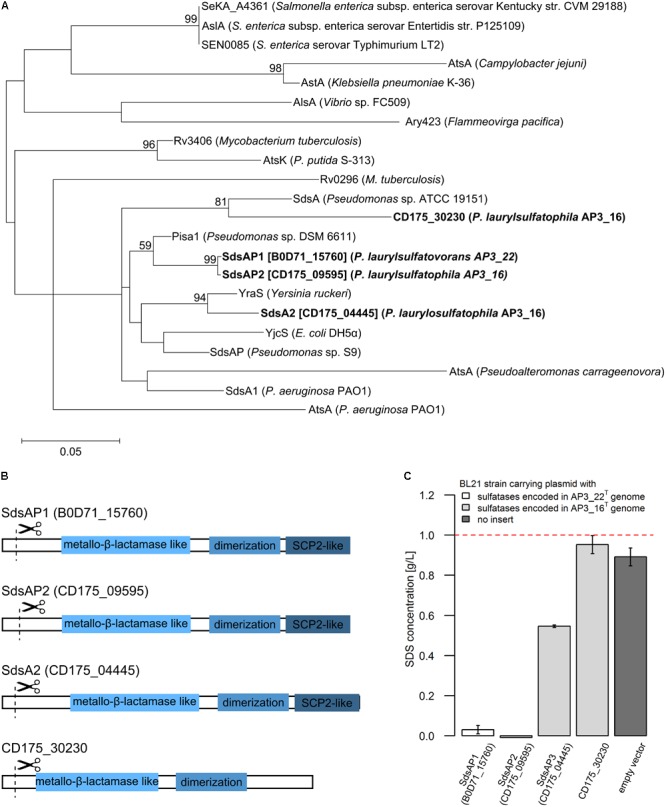
**(A)** Phylogenetic tree of the putative alkylsulfatases involved in SDS decomposition encoded in the genomes of the *P. laurylsulfatophila* AP3_16^T^ and *P. laurylsulfatovorans* AP3_22^T^ strains (shown in bold) and the known representatives of three groups of bacterial sulfatases. The evolutionary history was inferred by using the Maximum Likelihood method based on the Le_Gascuel_2008 model using a discrete Gamma distribution (+G). The tree is drawn to scale, with branch lengths measured in the number of substitutions per site. **(B)** Scheme of the domain structure of the four alkylsulfatases belonging to the third group of sulfatases. Three domains are shown as rectangulars, the scissors mark the predicted cleavage site of the signal peptide. **(C)** Study of SDS degradation of the *Escherichia coli* BL21 strains harboring possible four alkylsulfatases belonging to the third group of sulfatases. The SDS concentration was measured after 18 h of incubation of the BL21 induced (with 0.5 mM IPTG) strains carrying the recombinant proteins with 0.1% SDS in LB medium. BL21 strain without any plasmid was used as a control. The dotted line represents the SDS concentration in the uninoculated medium. All measurements were made in triplicate.

A phylogenetic comparison of the four proteins belonging to the third group of sulfatases and known representatives of the bacterial sulfatases is presented in **Figure 3A**. Enzymes coded by sequences B0D71_15760 (from AP3_22^T^) and CD175_09595 (from AP3_16^T^) clustered with Pisa1 – a stereoselective inverting alkylsulfatase with preferred activity toward secondary sulfate esters (Schober et al., 2011). Whereas proteins CD175_04445 (AP3_16^T^) and CD175_30230 (AP3_16^T^) clustered with sulfatases YraS and SdsA, respectively – enzymes involved in SDS metabolism (Davison et al., 1992; Navais et al., 2014).

Further DELTA-BLAST (domain enhanced lookup time accelerated BLAST) analysis of identified putative sulfatases showed that CD175_04445, CD175_09595 and B0D71_15760 proteins contain three domains: the metallo-β-lactamase like domain, an SCP-2-like domain and an alkyl sulfatase dimerization domain. In the protein sequence of the putative sulfatase CD175_30230, there was no SCP-2-like domain, involved together with the metallo-β-lactamase-like domain in recruiting hydrophobic substrates by the enzymes and binding of the sterols (**Figure 3B**). Further analysis with the SignalP server showed that all of the four potential alkylsulfatases have an N-terminal signal peptide consisting of 23–26 amino acid residues, similar to other members of the third group of sulfatases (Hagelueken et al., 2006; Long et al., 2011).

### Protein Overexpression and Verification of Alkylsulfatase Activity

The enzymes from the third group of sulfatases (CD175_04445, CD175_09595, CD175_30230, and B0D71_15760) were cloned into the pET-His expression system, either including or excluding the N-terminal signal sequences, and expressed in the *E. coli* BL21 (RIL) as a C-terminally His-tagged recombinant proteins. Further, the potential activity toward SDS was verified using the BL21 strains in which protein expression had been induced with 0.5 mM IPTG and cultures were incubated in the presence of SDS in the growth medium. The measurement of SDS concentration showed sulfatase activity in the three strains carrying the pET derivatives expressing enzyme with the N-terminal signal sequence – CD175_04445, CD175_09595 (originates from AP3_16 genome) and B0D71_15760 (from AP3_22^T^ genome) (**Figure 3C**). Lack of these signal peptides eliminated the ability of the strains to desulfurylate SDS. The *E. coli* strains expressing either B0D71_15760 or CD175_09595 recombinant protein presented a very similar level of detergent desulfurylation and decomposed 96.9 and 99.2% of the initial SDS concentration, respectively (measured after 24 h of incubation), whereas the strain expressing the putative sulfatase, CD175_04445 only degraded 45.4% of the surfactant.

The sulfatases B0D71_15760 and CD175_09595, designated as SdsAP1 and SdsAP2, respectively, are similar polypeptides with the same predicted length of 657 amino acids, pI of 5.91 and estimated molecular weights of 71.9 – 72.1 kDa. The enzyme CD175_04445 named SdsAP3, consists of 678 amino acids with molecular weight of 74.8 kDa and predicted pI of 6.01. SdsAP1 and SdsAP2 were successfully purified under native conditions from the soluble fractions after induction of *E. coli* BL21 (RIL) strains with IPTG. The monomeric molecular weight of the purified recombinant proteins was approximately 70 kDa on SDS-PAGE (**Supplementary Figure S4**). Despite many strategies tested for optimization of SdsAP3 overexpression (including different *E. coli* host strains, induction method and time) we were unable to obtain a protein fraction with acceptable homogeneity and activity. Consequently, only the SdsAP1 and SdsAP2 alkylsulfatases were further characterized.

### Biochemical Characterization of the Recombinant Sulfatases

**FIGURE 4:**
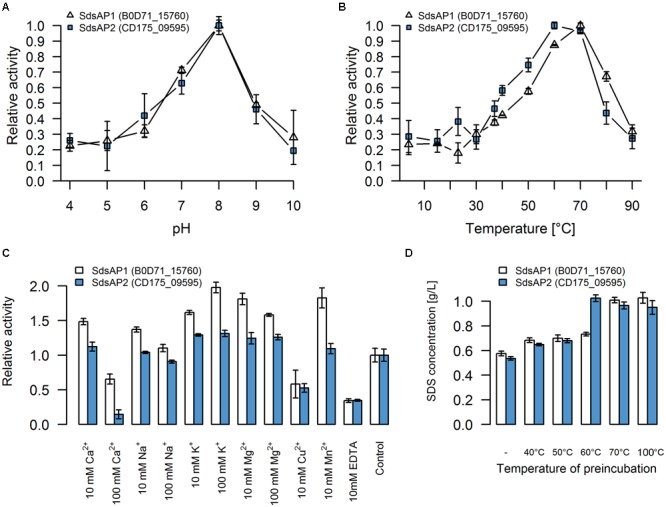
Properties of recombinant sulfatases. Effect of **(A)** pH, **(B)** temperature, **(C)** various additives, **(D)** preincubation in different temperatures on activity of the recombinant SdsAP1 (B0D71_15760 – shown in white) and SdsAP2 (CD175_09595 – shown in blue) sulfatases. pH profiles were measured at 37°C in 50 mM of different buffers: citrate buffer (pH 4.0–5.0), citrate-phosphate buffer (pH 6.0), Tris-HCl (pH 7.0 and 8.0), glycine-NaOH buffer (pH 9.0–10.0). Temperature profiles were measured at different temperatures (4–90°C) in 50 mM Tris-HCl buffer (pH 8.0). The effect of various ions (used as chloride salts) and EDTA was measured at 37°C in 50 mM Tris-HCl buffer (pH 8.0). The thermostability was tested by preincubation of the enzyme for 1 h in: 40, 50, 60 or 70°C or for 10 min in 100°C followed by standard activity testing measured at 37°C in 50 mM Tris-HCl buffer (pH 8.0). The values are means of the three replicates, and the error bars indicate the standard deviations.

For initial biochemical characterization, we tested the influence of temperature and pH on sulfatase activity. SdsAP1 and SdsAP2 both present the highest enzymatic activity toward SDS at similar pH (**Figure 4A**) with maximum activity at pH 8.0, but show differences in their temperature activity profiles (**Figure 4B**). The optimal temperature for SdsAP1 activity was 70°C whereas for SdsAP2 it was 60°C. Nevertheless, either SdsAP1 or SdsAP2 exhibit enzymatic activity in a broad range of tested temperatures.

The effect of various additives on sulfatase activity is presented in **Figure 4C**. These experiments showed that K^+^ and Mg^2+^ (in either 10 mM or 100 mM concentration) increased activity of both SdsAP1 and SdsAP2 alkylsulfatases, whereas they are inhibited by Cu^2+^, EDTA and Ca^2+^ in 100 mM concentrations. Analyzed enzymes differ in sensitivity to Na^+^, Mn^2+^, and 10 mM Ca^2+^. The activity of SdsAP1 increased in the presence of 10 mM Na^+^, however, 100 mM Na^+^ did not affect enzyme activity. Neither of the tested Na^+^ concentrations affected SdsAP2. Also, Mn^2+^ did not influence the SdsAP2 activity, although it had positive effect on SdsAP1, similar to effect caused by 10 mM Ca^2+^.

Thermostability was tested by preincubation of the sulfatases at 40, 50, 60, and 70°C for 1 h or 100°C for 10 min. Both of the proteins retained the enzymatic activity after preincubation in 40 and 50°C. However, in each case, enzymatic activity was decreased when compared to the control. Only the SdsAP1 protein was able to hydrolyze the SDS after 1 h of incubation at 60°C resulting in degradation up to 27% of the initial SDS concentration (**Figure 4D**). None of the tested proteins presented enzymatic activity after incubation at 70°C and above.

## Discussion

### Phylogenetic, Physiological and Biochemical Analyses and Comparative Genomics of the Selected Bacterial Strains

Genomic sequencing and analysis has become one of the basic research methods allowing to better understand metabolic potential of microorganisms but has also become one of the main elements of bacterial classification. Phylogenetic classification of newly isolated AP3_16 bacterial strain (Furmanczyk et al., 2017c) was performed according to standard methodology including both multi-genic approach (MLSA) and whole genome comparison.

Using the MLSA approach we showed that the AP3_16 isolate is closely related to *P. jessenii* DSM 17150^T^. However, ANIb and dDDH analyses based on the whole genome sequence comparison revealed that the AP3_16 strain shares the highest similarity to *P. laurylsulfatovorans* AP3_22^T^ - another member of the *P. jessenii* subgroup. This result is corroborated by the fact that AP3_16 shares more physiological and biochemical features with *P. laurylsulfatovorans* AP3_22^T^ (108 identical results in 118 tests) than with *P. jessenii* DSM 17150^T^ (98 identical results) or any other used type strain (100 identical results for *P. vancouverensis* DSM 17555^T^ and 98 identical results for *P. umsongensis* DSM 16611^T^). Furthermore, the highest calculated similarity levels (91.15% for ANIb and 46% for dDDH) show that AP3_16 is more divergent from any other type strain. According to the recent guidelines for establishment of novel bacterial species (Chun et al., 2018) AP3_16 strain should be classified as novel species for which we propose the name *P. laurylsulfatophila* sp. nov. with AP3_16^T^ as a type strain.

It is noteworthy that the GO enrichment analysis found the highest similarity between the two SDS degrading strains *P. laurylsulfatophila* AP3_16^T^ and *P. laurylsulfatovorans* AP3_22^T^, but also indicated their close relation to *P. jessenii* DSM 17150^T^, as they share 151 unique gene clusters. These 151 features are related to various transporters rather than to particular catabolic processes. Moreover, the analysis demonstrated specific functional similarity between the genomes of the three SDS degrading strains. *P. laurylsulfatophila* AP3_16^T^, *P. laurylsulfatovorans* AP3_22^T^, and *P. vancouverensis* DSM 17555^T^ share gene clusters related to alkanesulfonate monooxygenase activity and bacterial cell wall components, lipopolysaccharide and lipid A biosynthesis. These functional similarities between the two characterized here isolates (*P. laurylsulfatophila* AP3_16^T^ and *P. laurylsulfatovorans* AP3_22^T^) may reflect biochemical adaptation caused by environmental factors. These strains were isolated from peaty soil, sampled from a surface flow constructed wetland of a wastewater treatment plant operated by a pesticide packing company. This habitat was continuously exposed to various xenobiotics, including pesticides and detergents, especially anionic surfactants like SDS. Therefore, survival in such harsh conditions would require adaptations regarding specific cell wall structure or an efficient detergent degrading enzymatic apparatus.

Intriguingly, the genome of *P. umsongensis* DSM 16611^T^ contains genes with potential alkanesulfonate monooxygenase activity. Another member of this species – strain Gwa3 was described to have the ability to degrade hydrocarbons, which is often mediated by oxygenases and enhanced by the presence of surfactants (Pham et al., 2014). This could explain the increased presence of these genes in the mentioned genome. However, in our experiments, *P. umsongensis* DSM 16611^T^ could not degrade SDS, suggesting that proteins coded by potential alkanesulfonate monooxygenase genes could be non-functional or inactive toward SDS.

### Transcriptomic Analysis

The transcriptomic analysis described here is the first differential expression analysis showing the time-lapse response for exposure of a bacterial strain to SDS during the reported degradation of the detergent. However, similar single-time-point studies were performed for *P. aeruginosa* PAO1. This strain during growth with SDS tends to form macroscopic cell aggregates consisting of respiring cells embedded in an extracellular matrix (Klebensberger et al., 2006). To investigate and identify the molecular mechanisms involved in the SDS-induced aggregation the microarray analysis was used. The analysis of the differences in gene expression after 1 h of treatment with detergent led to the identification of *siaABCD* genes which are essential for the auto-aggregation process. Although the research was focused mainly on the stress response also some genes involved in the degradation of SDS were proposed, including the *sdsA1* (coding alkylsulfatase) or putative dehydrogenases (PA0364 and PA0366) possibly responsible for further oxidation steps after the hydrolysis of SDS to dodecanol (Klebensberger et al., 2009). These suggestions were recently confirmed by mutants analysis and resulted in identification of *laoABC* gene cluster coding long-chain alcohols oxidation system (Panasia and Philipp, 2018).

In contrast, our studies present the continuous dynamic response of the AP3_22^T^ strain toward SDS from the first contact with the detergent to the almost-finished degradation process. The results showed that although SDS could be used as a carbon source, in the first place it influences the integrity of cell envelopes and induces a global stress response combined with cell wall modification and repair. In the case of the AP3_22^T^ strain, this effect was observed for at least the first 30 min after contact with detergent. In contrary to *P. aeruginosa* PAO1 (Klebensberger et al., 2009), the AP3_22^T^ strain did not induce cell aggregation while grown with SDS, also the *siaABCD* genes or similar were not present in its genome. Furthermore, the elevated expression of genes encoding enzymes involved in synthesis of the cell membrane components like cardiolipin or other phospholipids suggest that SDS induces dynamic changes in the composition of cell membranes. Similarly, a study on the modifications of membrane lipids of *P. putida* A ATCC 12633 caused by tetradecyltrimethylammonium bromide (a cationic surfactant) revealed changes in the composition of phosphatidic acid, phosphatidylglycerol, and cardiolipin after exposure to detergent, but also increases ratio of saturated to unsaturated fatty acid (Boeris et al., 2007; Heredia et al., 2014). Since membrane fluidity is highly enhanced by the presence of the unsaturated fatty acids, their further production in the presence of SDS could kill cells. Reduction in the proportion of unsaturated fatty acids increases membrane rigidity and could help the cell survive in the presence of the detergent. These results suggest that a modulation of membrane composition could be the first alternative, adaptative step in response to detergent exposure and could be responsible for resistance to high surfactant concentrations. Interestingly, regarding species of *Pseudomonas*, the level of cardiolipin decreases after exposure to cationic detergents (Boeris et al., 2007), while our results suggest that it could be increased when in contact with anionic detergents like SDS.

As the second response to the SDS, the metabolism of the AP3_22^T^ strain shifted from lipid biosynthesis to the lipid catabolism with the onset of SDS degradation. Similar changes in expression levels of genes with assigned function connected with lipid catabolism were observed for the *P. aeruginosa* PAO1 using the microarray assay (Klebensberger et al., 2009; Panasia and Philipp, 2018). However, hardly none of present homologs of differentially expressed genes from *P. aeruginosa* PAO1, were significantly changed in AP3_22^T^ strain (data not shown). It suggests that either the regulation of these genes differs between these two strains or that other genes with similar functions could be involved in the SDS degradation in AP3_22^T^. At the end of the experiment, where the detergent was almost fully degraded, and nutrient limitation likely began, the AP3_22^T^ strain shifted its metabolism once again and presumably entered the stationary phase. Interestingly, genes encoding potential sulfatases from the AP3_22^T^ genome were never significantly regulated, which corresponds with our previous results (Furmanczyk et al., 2017c), suggesting that alkylsulfatase from this strain is constitutively expressed. Also the *laoABCR* homologous gene cluster (B0D71_16575-B0D71_16590), coding a system for the oxidation of long-chain alcohols in *P. aeruginosa* PAO1, was identified in AP3_22^T^ but not differentially expressed during the tested conditions.

### Alkylsulfatases – Enzymes Involved in SDS Desulfurization

Presence of functional alkylsulfatases in bacterial cells seems to be the most important feature for successful SDS desulfurylation and subsequent degradation. Known reports suggest that microorganisms degrading SDS could produce single (Hagelueken et al., 2006; Chaturvedi and Kumar, 2010, 2011; Jovcic et al., 2010) or multiple alkylsulfatases, for example *Pseudomonas* C12B produces five enzymes and *P. putida* FLA, six enzymes (Lillis et al., 1983). The two efficient SDS degraders from our previous report exhibit one or two bands with activity toward SDS on zymography gels (Furmanczyk et al., 2017c). Therefore, one of the main goal of this study was to investigate which of the sulfatases encoded by the AP3_16^T^ and AP3_22^T^ genomes are responsible for SDS desulfurylation and could be potentially used for development of biochemical tools to manage SDS in the environment. Detailed analysis of the genome sequences of these two *Pseudomonas* strains showed that they encode several sulfatases (6 for AP3_22^T^ and 15 or more for AP3_16^T^), which could be involved in detergent metabolism. As mentioned above, sulfatases are very heterogeneous and are divided into three main groups. Members of two of them – the Fe(II) dependent dioxygenases (group II) and especially alkylsulfatases (group III) – are well known for SDS decomposition (Kahnert and Kertesz, 2000; Müller et al., 2004; Hagelueken et al., 2006; Long et al., 2011). According to our results, based on the conserved domain search of the proteins annotated as sulfatases, genomes of the detergent degraders characterized here encode sulfatases belonging only to the arylsulfatases (group I) or the alkylsulfatases (group III). Simple cloning of these genes into *E. coli* led to the identification of two strains with SDS degrading abilities carrying the enzymes affiliated with group III bacterial sulfatases, which strongly suggests the importance of alkylsulfatases in SDS metabolism. Detailed analysis of the proteins belonging to group III showed that all of the potential enzymes contain a signal sequence and three of four of the proteins possessing a set of domains typical for alkylsulfatases (the metallo-β-lactamase like domain, SCP-2-like domain and alkyl sulfatase dimerization domain) (Hagelueken et al., 2006; Long et al., 2011). The fourth protein (CD175_30230) lacks the SCP-2-like domain, which is postulated to be involved in substrate binding (Hagelueken et al., 2006). Experimental verification confirmed the functionality of the three enzymes (containing all of the three domains) in *E. coli* – two encoded in the AP3_16^T^ genome (SdsAP2, SdsAP3) and one originating from AP3_22^T^ genome (SdsAP1). However, the CD175_30230 protein presented no activity toward SDS while expressed in *E. coli*. This strongly emphasizes the important role of the SCP-2-like domain during recruiting especially long aliphatic substrates like SDS, for sulfate ester bond cleavage. The strains harboring the functional alkylsulfatases differ significantly in their ability for SDS degradation. This may be a result of divergent enzymatic activity but also the level of protein expression or incorrect protein folding. Further problems with optimization of CD175_30230 expression confirmed these observations.

The two purified and characterized here proteins, SdsAP1 and SdsAP2, share high amino acid identity (93%) and according to the phylogenetic analysis, show the closest similarity to the Pisa1 protein – a stereoselective inverting alkylsulfatase with activity preference toward secondary sulfate esters (Schober et al., 2011). The Pisa1 was identified in *Pseudomonas* sp. DSM 6611 isolated from soil for its ability to degrade halogenated aromatic compounds (Oltmanns et al., 1989).

Nevertheless, concerning the properties of the recombinant SdsAP1 and SdsAP2, they show high similarity to SdsAP, a thermostable alkylsulfatase from *Pseudomonas* sp. S9 (Long et al., 2011). This enzyme presents maximum activity at pH 9.0 and 70°C, similar to SdsAP1 (pH 8.0 and 70°C) and SdsAP2 (pH 8.0 and 60°C). However, the enzymes described here were less stable in elevated temperatures than SdsAP. Moreover, enzymatic activities differ in the presence of various ions in the reaction buffer. Activity of SdsAP1 and SdsAP2 sulfatases increased in presence of K^+^, Mg^2+^ and low concentrations (10 mM) of Ca^2+^ and Na^+^. However, 100 mM of the latter two elements inhibited activity of the analyzed enzymes. In contrast, as shown by Long et al. (2011) these additives did not have a significant impact on the activity of SdsAP. Also Mn^2+^ had the opposite effect on the characterized sulfatases and SdsAP (Long et al., 2011). All of the enzymes, including the SdsAP, were inhibited by the Cu^2+^ and EDTA. The effect caused by the chelator suggests that similar to SdsAP, SdsAP1 and SdsAP2 could require metal ions as cofactors for catalysis.

## Conclusion

In this study, we characterized two efficient SDS degrading strains isolated from a peaty soil from biological wastewater treatment plant. These microorganisms, named AP3_16 and AP3_22, belonging to the *P. jessenii* subgroup, represent previously identified efficient surfactant SDS degraders differing in the resistance toward SDS and their enzymatic apparatus involved in detergent desulfurization. A comparison of genome sequences of these isolates and the GO enrichment analysis showed that the genomes of SDS-degraders encode more genes connected with biosynthesis of bacterial cell walls and alkanesulfonate monooxygenase activity than their closest relatives from *Pseudomonas*. This could be an adaptation to living in an environment exposed to detergents. The transcriptomic analysis of the response of *P. laurylsulfatovorans* AP3_22^T^ to detergent also supports this hypothesis. Furthermore, we identify three novel enzymes with activity toward SDS belonging to the third class of sulfatases. Purification and preliminary description of the two most promising recombinant proteins shed light on the SDS desulfurization process and enlarge the portfolio of potentially useful biotools for environment bioremediation. However, during future alkylsulfatase characterization, including substrate specificity, it would be helpful to better understand the enzymatic reaction catalyzed by the third group of bacterial sulfatases.

### Description of *P. laurylsulfatophila* sp. nov.

*Pseudomonas laurylsulfatophila* sp. nov. [lau.ryl.sul.fa.to’phi.la N.L. n. laurylsulfatas, lauryl sulfate; N.L. fem. adj. phila (from Gr. adj. philos), friendly to, loving; N.L. fem. adj. laurylsulfatophila, lauryl sulfate-loving].

Cells are Gram-negative, approximately 0.6 × 1.9 μm in size, motile by a single polar flagellum. Colonies are smooth, round and milky on Lysogeny Broth (LB) agar. Cells produce a fluorescent pigment on King B medium. Catalase- and oxidase-positive but urease, arginine dehydrolase and β-galactosidase negative. They do not reduce nitrates and do not hydrolyze esculin and gelatin. Indole is not produced and there is no glucose fermentation. *P. laurylsulfatophila* can grow at the temperature ranging from 8 to 42°C but not at 4°C. Optimum pH for growth was pH 7.0, however, the strain was able to grow in pH ranging from 5 to 10. Moreover, strain has the ability to grow in media supplemented with up to 7% NaCl but not at higher salinity. Analysis performed using Biolog System GENIII plate test indicated that the strain has the ability to oxidize variety of substrates: dextrin, sucrose, *N*-acetyl-D-glucosamine, A-D-glucose, D-mannose, D-fructose, D-galactose, D-fucose, L-fucose, D-mannitol, D-arabitol, glycerol, D-fructose-6-PO_4_, D-serine, L-alanine, L-arginine, L-aspartic acid, L-glutamic acid, L-histidine, L-pyroglutamic acid, L-serine, pectin, D-galacturonic acid, L-galacturonic acid lactone, D-gluconic acid, D-glucuronic acid, glucuronamide, mucic acid, quinic acid, D-saccharic acid, methyl pyruvate, L-lactic acid, citric acid, α -keto-glutaric acid, D-malic acid, L-malic acid, bromo-succinic acid, Tween 40, Γ-amino-butyric acid, α-hydroxy-butyric acid, β -hydroxy-D, L-butyric acid, α -keto-butyric acid, acetoacetic acid, propionic acid, acetic acid, formic acid. Other organic substrates included in Biolog GENIII microplates are not oxidized. The *P. laurylsulfatophila* sp. nov. can use sodium dodecyl sulfate as a sole carbon source. The test using API20 NE strip shows that the strain assimilate: D-glucose, D-mannose, D-mannitol, *N*-acetyl-glucosamine, potassium gluconate, capric acid, malic acid, trisodium citrate and phenylacetic acid and do not assimilate d-maltose and adipic acid. The G + C content of the DNA of AP3_16^T^ is 60.1 mol%. The type strain is AP3_16^T^ (=DSM 105097^T^ = PCM 2903^T^). Digital Protologue Taxonumber: TA00573.

## Author Contributions

AS and LL conceived and directed the studies. EF performed the bacterial phenotype analysis, DNA and RNA isolation, DNA and RNA libraries preparation, all bioinformatics analysis, protein overexpression, and purification and enzymatic assays. EF wrote the manuscript, consulted and corrected by AS, LL, and AD. LL and AD provided the funding for this work. All authors read and approved the final manuscript.

## Conflict of Interest Statement

The authors declare that the research was conducted in the absence of any commercial or financial relationships that could be construed as a potential conflict of interest.

## Footnotes

1 http://www.cbs.dtu.dk/services/SignalP/
