# Supplementary material for: Genomic and Functional Characterization of Environmental Strains of SDS-Degrading Pseudomonas spp., Providing a Source of New Sulfatases
Source: Front Microbiol. 2018 Aug 17;9:1795. doi: 10.3389/fmicb.2018.01795 (PMC6107682; doi:10.3389/fmicb.2018.01795)
Supplement: Supplementary file 1 [file Data_Sheet_1.docx]

*Supporting Information for*

Genomic and functional characterization of environmental strains of SDS-degrading *Pseudomonas* spp., providing a source of new sulfatases

**Ewa M. Furmanczyk^1^, Leszek Lipinski^1^, Andrzej Dziembowski^1,2^, Adam Sobczak^1,2^***

^1^ Institute of Biochemistry and Biophysics, Polish Academy of Sciences, Warsaw, Poland

^2^ Institute of Genetics and Biotechnology, Faculty of Biology, University of Warsaw, Poland

*Corresponding author at: Institute of Biochemistry and Biophysics, Polish Academy of Sciences, Pawinskiego 5a, 02-106 Warsaw, Poland

E-mail address: adams@ibb.waw.pl

Tel: +48 22 592 57 78

Fax: +48 22 592 21 90


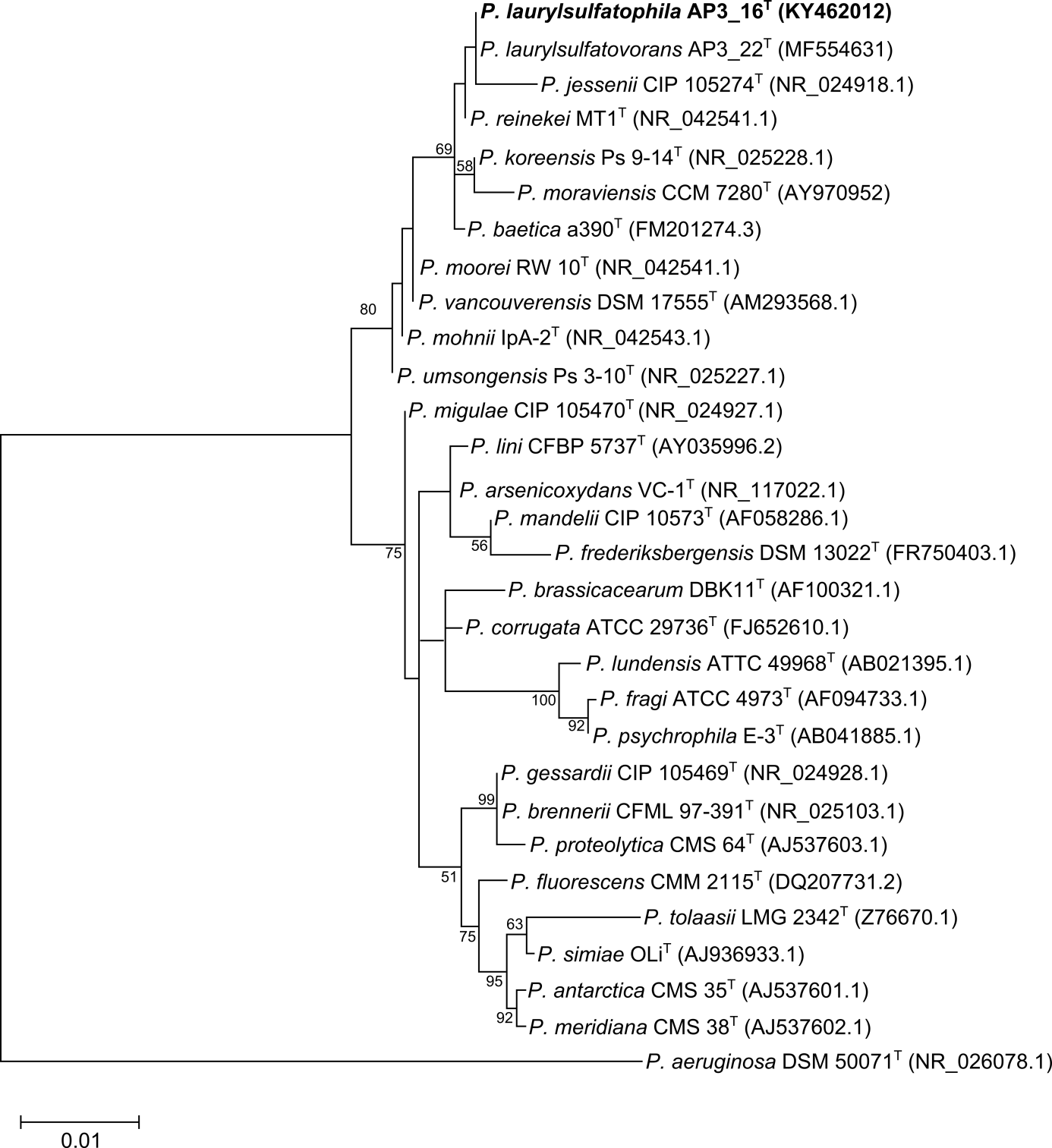


**Figure S1.** Phylogeny of type strains closely related to *Pseudomonas laurylsulfatophila* AP3_16^T^ strain (in bold) based on 16S rRNA gene sequence. All positions containing gaps or missing data were eliminated, which resulted in a 1,297 bp sequence in the final dataset. Bootstrap values are represented at the branching points (only values >50% are shown). The bar represents 0.01 substitutions per site. Accession numbers of sequences used in this analysis are in parentheses.


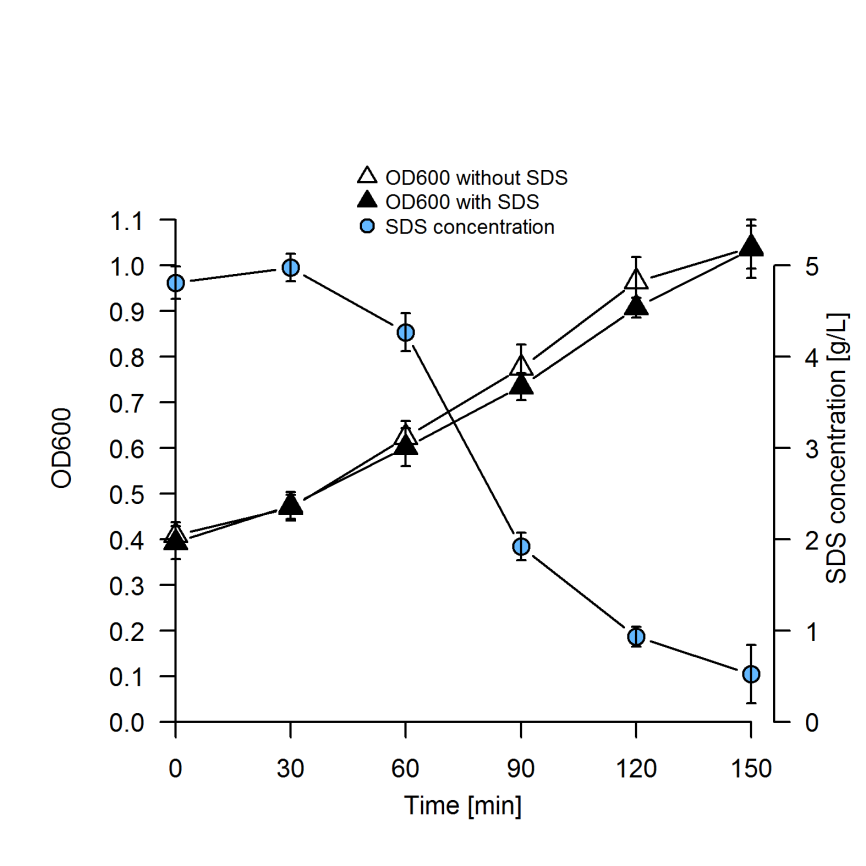


**Figure S2**. Growth rate and SDS degradation degree during the RNA-seq experiment. *P. laurylsulfatovorans* AP3_22^T^ strain was cultured in 0.1 LB medium with 5 g/L SDS (or without detergent as a control). The optical density of the cultures (illustrated as white and black triangles) and the SDS concentration (blue circles) were measured every 30 minutes. The values are means of three replicates and the error bars indicate the standard deviations.

**
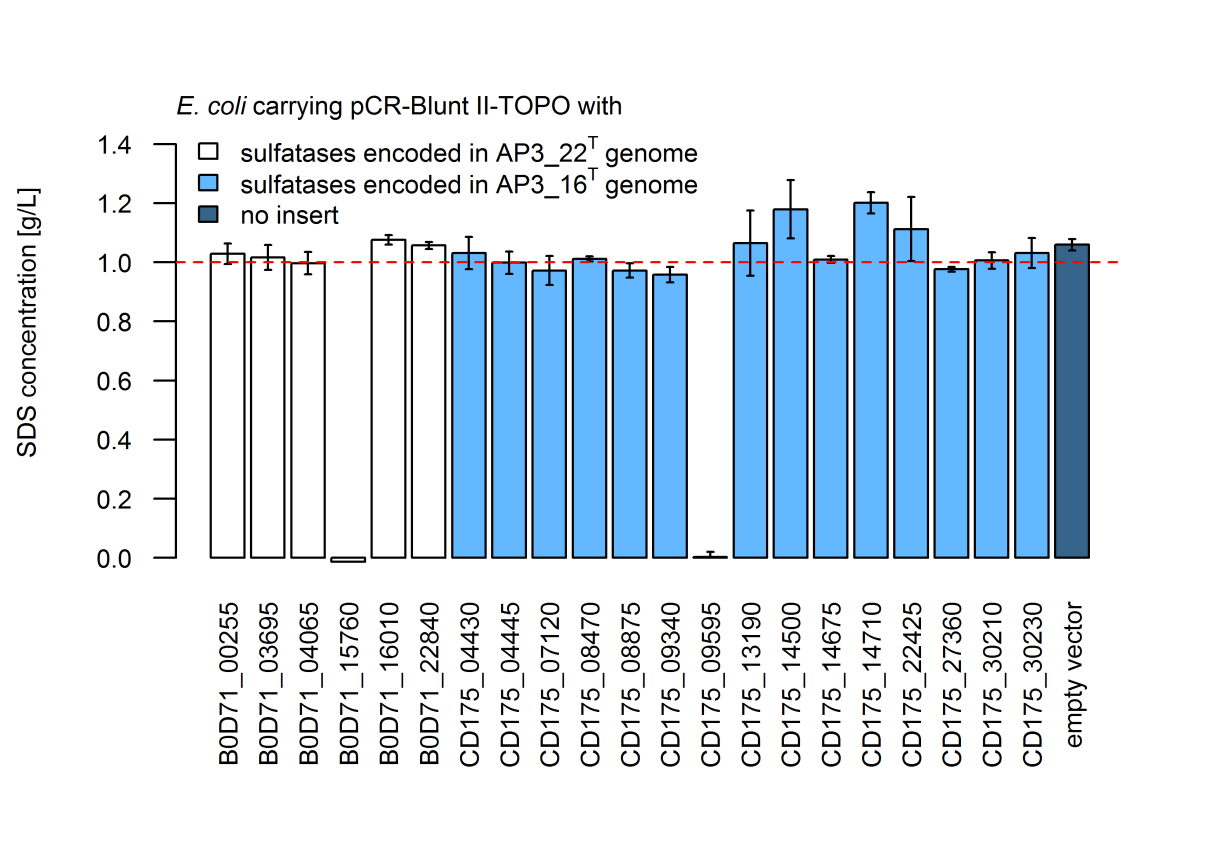
**

**Figure S3**. Comparison of degradation capabilities of *E. coli* TOP10 carrying pCR-Blunt-II TOPO vectors derives with different genome fragments coding the potential alkylsulfatases. The strains were cultured in LB medium with 0.1% SDS. The detergent concentration was verified with colorimetric assay after 24 hours of incubation. Strain carrying empty vector was used as a control. The values are means of the three replicates, and the error bars indicate the standard deviations. The dotted line represents the SDS concentration at the beginning of the experiment.

**
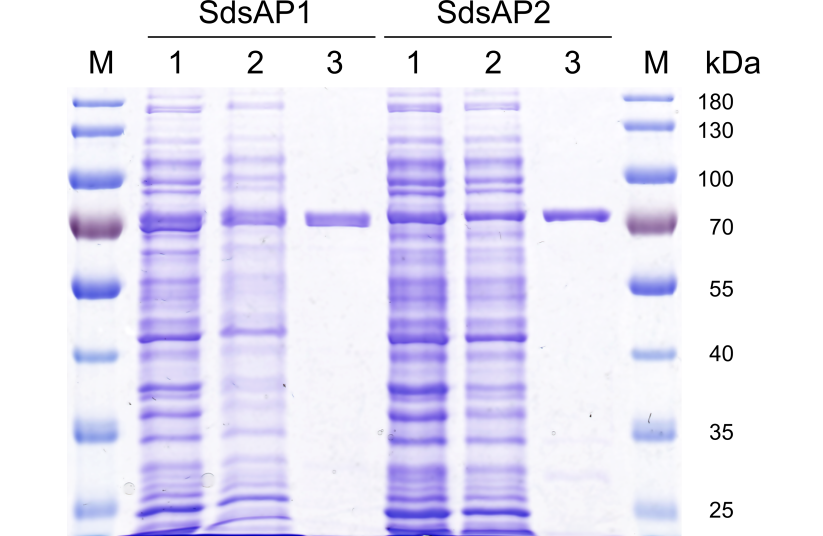
**

**Figure S4.** SDS-PAGE analysis of recombinant SdsAP1 and SdsAP2 proteins. M - molecular mass marker, lanes: 1, total protein extracts from cells induced with 0.5 mM IPTG; 2, soluble fractions after bacteria disruption; 3, purified proteins eluted from Protino Ni-IDA.

**Table S1.** Accession numbers of the sequences of different *Pseudomonas* spp. strains used in the MLSA phylogenetic analysis.

| **Species** | **Gene name** | **Accession number** | **Strain designation** | **Species** | **Gene name** | **Accession number** | **Strain designation** | **Species** | **Gene name** | **Accession number** | **Strain designation** |
| --- | --- | --- | --- | --- | --- | --- | --- | --- | --- | --- | --- |
| *P. jessenii* | 16S rRNA | AF068259.1 | CIP 105274^T^ | *P. lini* | 16S rRNA | AY035996.2 | CFBP 5737^T^ | *P. fluorescens* | 16S rRNA | DQ207731.2 | CCM 2115^T^ |
|  | *gyrB* | AM293562.1 | DSM 17150^T^ |  | *gyrB* | FN554196.1 | CIP 107460^T^ |  | *gyrB* | D86016.1 | IAM 12022^T^ |
|  | *rpoB* | AJ717447.1 | CIP 105274^T^ |  | *rpoB* | AJ717466.1 | CIP 107460^T^ |  | *rpoB* | AJ717451.1 | CIP 69.13^T^ |
|  | *rpoD* | FN554473.1 | CIP 105274^T^ |  | *rpoD* | FN554478.1 | CIP 107460^T^ |  | *rpoD* | D86033.1 | IAM 12022^T^ |
| *P. moorei* | 16S rRNA | AM293566.1 | RW 10^T^ | *P. mandelii* | 16S rRNA | AF058286.1 | CIP 105273^T^ | *P. tolaasii* | 16S rRNA | Z76670.1 | LMG 2342^T^ |
|  | *gyrB* | AM293560.1 | RW 10^T^ |  | *gyrB* | FN554199.1 | LMG 21607^T^ |  | *gyrB* | AB039423.1 | NCPPB 2192^T^ |
|  | *rpoB* | FN554742.1 | CCUG 53114^T^ |  | *rpoB* | AJ717435.1 | CIP 105273^T^ |  | *rpoB* | AJ717467.1 | LMG 2342^T^ |
|  | *rpoD* | FN678363.1 | DSM 12647^T^ |  | *rpoD* | FN554482.1 | LMG 21607^T^ |  | *rpoD* | AB039561.1 | NCPPB 2192^T^ |
| *P. mohnii* | 16S rRNA | AM293567.1 | IpA-2^T^ | *P. arsenicoxydans* | 16S rRNA | FN645213.1 | VC-1^T^ | *P. simae* | 16S rRNA | AJ936933.1 | OLi^T^ |
|  | *gyrB* | AM293561.1 | IpA-2^T^ |  | *gyrB* | HE800469.1 | CECT 7543^T^ |  | *gyrB* | FN554227.1 | CCUG 50988^T^ |
|  | *rpoB* | FN554741.1 | CCUG 53115^T^ |  | *rpoB* | HE800503.1 | CECT 7543^T^ |  | *rpoB* | FN554757.1 | CCUG 50988^T^ |
|  | *rpoD* | FN554487.1 | CCUG 53115^T^ |  | *rpoD* | HE800488.1 | CECT 7543^T^ |  | *rpoD* | FN554513.1 | CCUG 50988^T^ |
| *P. vancouverensis* | 16S rRNA | AM293568.1 | DSM 17555^T^ | *P. frederiksbergensis* | 16S rRNA | FR750403.1 | DSM 13022^T^ | *P. antarctica* | 16S rRNA | AJ537601.1 | CMS 35^T^ |
|  | *gyrB* | AM293558.1 | DSM 17555^T^ |  | *gyrB* | AM084676.1 | DSM 13022^T^ |  | *gyrB* | FN554169.1 | LMG 22709^T^ |
|  | *rpoB* | AJ717473.1 | CIP 106707^T^ |  | *rpoB* | AJ717465.1 | CIP 106887^T^ |  | *rpoB* | FN554727.1 | LMG 22709^T^ |
|  | *rpoD* | FN554517.1 | ATCC 700688^T^ |  | *rpoD* | AM084335.2 | DSM 13022^T^ |  | *rpoD* | FN554450.1 | LMG 22709^T^ |
| *P. umsongensis* | 16S rRNA | AF468450.1 | Ps 3-10^T^ | *P. aeruginosa* | 16S rRNA | HE978271.1 | DSM 50071^T^ | *P. meridiana* | 16S rRNA | AJ537602.1 | CMS 38^T^ |
|  | *gyrB* | AM293564.1 | DSM 16611^T^ |  | *gyrB* | AJ633104.1 | CCM 1960^T^ |  | *gyrB* | FN554203.1 | CIP 108465^T^ |
|  | *rpoB* | FN554763.1 | LMG 21317^T^ |  | *rpoB* | AJ717442.1 | LMG 1242^T^ |  | *rpoB* | FN554740.1 | CIP 108465^T^ |
|  | *rpoD* | FN554516.1 | LMG 21317^T^ |  | *rpoD* | AJ633568.1 | CCM 1960^T^ |  | *rpoD* | FN554485.1 | CIP 108465^T^ |
| *P. reinekei* | 16S rRNA | AM293565.1 | MT1^T^ | *P. gessardii* | 16S rRNA | AF074384.1 | CIP 105469^T^ | *P. lundensis* | 16S rRNA | AB021395.1 | ATCC 49968^T^ |
|  | *gyrB* | AM293559.1 | MT1^T^ |  | *gyrB* | FN554186.1 | CIP 105469^T^ |  | *gyrB* | FN554197.1 | LMG 13517^T^ |
|  | *rpoB* | FN554754.1 | CCUG 53116^T^ |  | *rpoB* | AJ717438.1 | CIP 105469^T^ |  | *rpoB* | AJ717428.1 | CIP 103272^T^ |
|  | *rpoD* | FN678362.1 | DSM 18361^T^ |  | *rpoD* | FN554468.1 | CIP 105469^T^ |  | *rpoD* | FN554479.1 | LMG 13517^T^ |
| *P. koreensis* | 16S rRNA | KU041145.1 | Ps 9-14^T^ | *P. brennerii* | 16S rRNA | AF268968.1 | CFML 97-391^T^ | *P. fragi* | 16S rRNA | AF094733.1 | ATCC 4973^T^ |
|  | *gyrB* | AM293563.1 | DSM 16610^T^ |  | *gyrB* | FN554176.1 | DSM 15294^T^ |  | *gyrB* | DQ887266.1 | ATCC 4973^T^ |
|  | *rpoB* | FN554737.1 | LMG 21318^T^ |  | *rpoB* | AJ717482.1 | CIP 106646^T^ |  | *rpoB* | AJ717444.1 | LMG 2191^T^ |
|  | *rpoD* | FN554476.1 | LMG 21318^T^ |  | *rpoD* | FN554457.1 | DSM 15294^T^ |  | *rpoD* | FN554466.1 | ATCC 4973^T^ |
| *P. moraviensis* | 16S rRNA | AY970952.1 | CCM 7280^T^ | *P. proteolytica* | 16S rRNA | AJ537603.1 | CSM 64T | *P. psychrophila* | 16S rRNA | AB041885.1 | E-3^T^ |
|  | *gyrB* | FN554206.1 | DSM 16007^T^ |  | *gyrB* | FN554220.1 | CIP 108464^T^ |  | *gyrB* | FN554221.1 | DSM 17535^T^ |
|  | *rpoB* | FN554743.1 | DSM 16007^T^ |  | *rpoB* | FN554752.1 | CIP 108464^T^ |  | *rpoB* | AJ717464.1 | CIP 107901^T^ |
|  | *rpoD* | FN554490.1 | DSM 16007 |  | *rpoD* | FN554505.1 | CIP 108464^T^ |  | *rpoD* | FN554506.1 | DSM 17535^T^ |
| *P. baetica* | 16S rRNA | FM201274.3 | a390^T^ | *P. brassicacearum* | 16S rRNA | AF100321.1 | DBK11^T^ | *P. corrugata* | 16S rRNA | D84012.1 | ATCC 29736^T^ |
|  | *gyrB* | FM201278.1 | a390^T^ |  | *gyrB* | AM084675.1 | CFBP 11706^T^ |  | *gyrB* | AB039460.1 | NCPPB 2445^T^ |
|  | *rpoB* | HE800504.1 | CECT 7720^T^ |  | *rpoB* | AJ717436.1 | CIP 107059^T^ |  | *rpoB* | AJ717487.1 | LMG 2172^T^ |
|  | *rpoD* | FN678357.1 | a390^T^ |  | *rpoD* | AM084334.1 | CFBP 11706^T^ |  | *rpoD* | AB039566.1 | NCPPB 2445^T^ |
| *P. migulae* | 16S rRNA | AF074383.1 | CIP 105470^T^ | P. laurylsulfatovorans | 16S rRNA | MF554631 | AP3_22^T^ | *P. lurylsulfatophila* AP3_16^T^ | 16S rRNA | KY462012 | |
|  | *gyrB* | FN554204.1 | CCUG 43165^T^ |  | *gyrB* | MF673736 | AP3_22^T^ |  | *gyrB* | Sequences received from the draft genome sequence -accession number - NIRS00000000 | |
|  | *rpoB* | AJ717446.1 | CIP 105470^T^ |  | *rpoB* | MF673733 | AP3_22^T^ |  | *rpoB* |  |  |
|  | *rpoD* | FN554486.1 | CCUG 43165^T^ |  | *rpoD* | MF673738 | AP3_22^T^ |  | *rpoD* |  |  |

**Table S2.** Primers used in the study and the detailed PCR conditions.

| Primer name | Primer sequence [5'-3'] | Product size [bp] | Product name | Amplification time |
| --- | --- | --- | --- | --- |
| AP3_16SUL_1F | ACGCCTGTTTAATCGTGTCC | 2010 | CD175_04430 | 65 s |
| AP3_16SUL_1R | GCGCTGAAATGATGTGATCC |  |  |  |
| AP3_16SUL_2F | TGACAGACAGTCGCAGATCG | 1867 | CD175_07120 | 65 s |
| AP3_16SUL_2R | CAAGCCAGCTCCTACAGTTG |  |  |  |
| AP3_16SUL_3F | AGTCGGGCCATGTCTTGCTC | 1846 | CD175_08470 | 65 s |
| AP3_16SUL_3R | GGCCTGCCACCACAAAGTAG |  |  |  |
| AP3_16SUL_4F | GCGGTGCTGCATATCTGACG | 1815 | CD175_08875 | 65 s |
| AP3_16SUL_4R | ATTCAGGCGGCCTCGATACG |  |  |  |
| AP3_16SUL_5F | ATTCGACCGGCGCATTTACC | 1895 | CD175_09340 | 65 s |
| AP3_16SUL_5R | TGCAGATCGGGCAAGTGTTC |  |  |  |
| AP3_16SUL_6F | TCAAGGGTGGCTGGAAATAC | 2724 | CD175_14500 | 90 s |
| AP3_16SUL_6R | TGAGGGTCAAGGCAAATACG |  |  |  |
| AP3_16SUL_7F | CAGCAGCACTGCGAGAAGAC | 2008 | CD175_14675 | 65 s |
| AP3_16SUL_7R | TTCGGCCGTTATCGAGGAGG |  |  |  |
| AP3_16SUL_8F | GTAAACGCCTCTGTGGTTTC | 1850 | CD175_14710 | 65 s |
| AP3_16SUL_8R | TGGATTCGGCTGTTACTTGG |  |  |  |
| AP3_16SUL_10F | GGCGGATCAAACAAGTGGTC | 796 | CD175_27360 | 30 s |
| AP3_16SUL_10R | GTAGCGGATGCGTTCAAGTG |  |  |  |
| AP3_16SUL_11F | TGGACGCTCGACCCTTATTC | 1820 | CD175_30210 | 65 s |
| AP3_16SUL_11R | CTACAGAGGATCGCGTACAC |  |  |  |
| AP3_16SUL_12F | GTTCGGCGTCATCTCATAAC | 2272 | CD175_04445 | 90 s |
| AP3_16SUL_12R | TATTCAGGGTCGCGAAGTAG |  |  |  |
| AP3_16SUL_13F | GCAGCATTCGCCGACTAGAC | 2008 | CD175_30230 | 65 s |
| AP3_16SUL_13R | CCGGGCAAGGACAAGGAATC |  |  |  |
| AP3_16SUL_14F | GGCGATCAGGTGCAGCAAAG | 2321 | CD175_09595 | 90 s |
| AP3_16SUL_14R | CCCGCATCATCGTTGACCTC |  |  |  |
| AP3_16SUL_15F | TCCACGGTATAGCCTTTGAG | 2235 | CD175_13190 | 90 s |
| AP3_16SUL_15R | TTAACGTTCGCCGACTTGAC |  |  |  |
| AP3_16SUL_16F | AAGCGGCAGACCAAATCCTC | 1902 | CD175_22425 | 65 s |
| AP3_16SUL_16R | TGCGGAAGGTTCGGATGAAG |  |  |  |
| AP3_22SUL_1F | CGCCCTTGACCGTTATTCCC | 1853 | B0D71_00255 | 65 s |
| AP3_22SUL_1R | TGGCCGGATATCGGTTCCTC |  |  |  |
| AP3_22SUL_2F | GCCCTTGGCATGTTCTGGTATC | 1912 | B0D71_03695 | 65 s |
| AP3_22SUL_2R | TTTGCGGGTGCCTTGAGTAG |  |  |  |
| AP3_22SUL_3F | ACAGTCGCAGATCGGTCGTCTC | 1852 | B0D71_04065 | 65 s |
| AP3_22SUL_3R | ATAGGGCCGAAACGGTCCCAAG |  |  |  |
| AP3_22SUL_4F | TTTGCCACTGCCTGACTC | 2421 | B0D71_15760 | 65 s |
| AP3_22SUL_4R | GGCGGCATCACATTCAAC |  |  |  |
| AP3_22SUL_5F | ATTCGGCTGACTACTTGG | 1850 | B0D71_16010 | 65 s |
| AP3_22SUL_5R | CCCGGTAATGAAGACTGAG |  |  |  |
| AP3_22SUL_6F | GGCGGATCAAACAAGTGGTC | 1859 | B0D71_22840 | 65 s |
| AP3_22SUL_6R | CGCATCACATCGGTCATTGTAG |  |  |  |
| A16S12LF | GTTTAACTTTAAGAAGGAGATATACCATGGATGTACAACAAGACCCTCCATCT | 2095 | CD175_04445 full length | 65 s |
| A16S12R | GGTGACCCTGAAAATACAAATTCTCCTCGAGCTGCTGGGTGCCTGGCAGTA |  |  |  |
| A16S13LF | GTTTAACTTTAAGAAGGAGATATACCATGGATGCCGCGTTTCCCGCTGTCTCC | 1831 | CD175_30230 full length | 65 s |
| A16S13R | GGTGACCCTGAAAATACAAATTCTCCTCGAGGACCTTGTACGGATCGAACA |  |  |  |
| A16S14LF | GTTTAACTTTAAGAAGGAGATATACCATGGATGCCTCGTTTCACCTTGAG | 2032 | CD175_09555 full length | 65 s |
| A16S14R | GGTGACCCTGAAAATACAAATTCTCCTCGAGCGGGGTAACGATATTGAACT |  |  |  |
| A22S04LF | GTTTAACTTTAAGAAGGAGATATACCATGGATGCCTCGTTTCACCTTGAGCCCTCG | 2032 | B0D71_15760 full length | 65 s |
| A22S04R | GGTGACCCTGAAAATACAAATTCTCCTCGAGAGGCGTGACGATATTGAACTGCG |  |  |  |
| A16S12SF | GTTTAACTTTAAGAAGGAGATATACCATGGATGGCCGATGCAAACGGCGCAGCTTCC | 2026 | CD175_04445 without signal sequence | 65 s |
| A16S12R | GGTGACCCTGAAAATACAAATTCTCCTCGAGCTGCTGGGTGCCTGGCAGTA |  |  |  |
| A16S13SF | GTTTAACTTTAAGAAGGAGATATACCATGGATGGACGAGTCACTGTTCATGG | 1762 | CD175_30230 without signal sequence | 65 s |
| A16S13R | GGTGACCCTGAAAATACAAATTCTCCTCGAGGACCTTGTACGGATCGAACA |  |  |  |
| A16S14SF | GTTTAACTTTAAGAAGGAGATATACCATGGATGGTGGTCGCCGCCGATGCACC | 1966 | CD175_09555 without signal sequence | 65 s |
| A16S14R | GGTGACCCTGAAAATACAAATTCTCCTCGAGCGGGGTAACGATATTGAACT |  |  |  |
| A22S04SF | GTTTAACTTTAAGAAGGAGATATACCATGGATGGCCGATGCACCGGTCGCGGCCA | 1957 | B0D71_15760 without signal sequence | 65 s |
| A22S04R | GGTGACCCTGAAAATACAAATTCTCCTCGAGAGGCGTGACGATATTGAACTGCG |  |  |  |

**Table S3.** Physiological and biochemical characteristic of *P. laurylsulfatophila* AP3_16^T^ and the other closest *Pseudomonas* type strains. The strains were tested using Biolog GEN III Microplate, API20 NE strips and other tests characterized in the Materials and Methods section.

| **Carbon source** | **1** | **2** | **3** | **4** | **5** |
| --- | --- | --- | --- | --- | --- |
| Dextrin | + | - | - | - | - |
| D-Maltose | - | - | - | - | - |
| D-Trehalose | - | - | - | - | - |
| D-Cellobiose | - | - | - | - | - |
| Gentiobiose | - | - | - | - | - |
| Sucrose | + | + | - | - | - |
| D-Turanose | - | - | - | - | - |
| Stachylose | - | - | - | - | - |
| D-Raffinose | - | - | - | - | - |
| Α-D-Lactose | - | - | - | - | - |
| D-Melibiose | - | - | - | - | - |
| Β-Methyl-D-Glucoside | - | - | - | - | - |
| D-Salicin | - | - | - | - | - |
| N-Acetyl-D-Glucosamine | + | + | + | - | + |
| N-Acetyl-Β-D-Mannosamine | - | - | - | - | - |
| N-Acetyl-D-Galactosamine | - | - | - | - | - |
| N-Acetyl Neuraminic Acid | - | - | - | - | - |
| Α-D-Glucose | + | + | + | + | + |
| D-Mannose | + | + | + | + | + |
| D-Fructose | + | + | + | + | + |
| D-Galactose | + | + | + | + | - |
| 3-Methyl Glucose | - | - | - | - | - |
| D-Fucose | + | - | - | - | + |
| L-Fucose | + | - | - | - | + |
| L-Rhamnose | - | - | - | - | - |
| Inosine | - | - | - | - | + |
| D-Sorbitol | - | - | - | - | - |
| D-Mannitol | + | - | + | - | + |
| D-Arabitol | + | - | + | - | + |
| Myo-Inositol | - | - | - | - | - |
| Glycerol | + | + | + | + | + |
| D-Glucose-6PO_4_ | - | - | - | - | - |
| D-Fructose-6-PO_4_ | + | - | - | - | - |
| D-Aspartic Acid | - | - | + | - | - |
| D-Serine | + | + | + | - | + |
| Gelatin | - | - | - | - | - |
| Glycyl-L-Proline | - | - | + | + | - |
| L-Alanine | + | + | + | + | + |
| L-Arginie | + | + | + | + | + |
| L-Aspartic Acid | + | + | + | + | + |
| L-Glutamic Acid | + | + | + | + | + |
| L-Histidine | + | + | + | + | + |
| L-Pyroglutamic Acid | + | + | + | + | - |
| L-Serine | + | + | + | + | + |
| Pectin | + | + | + | + | - |
| D-Galacturonic Acid | + | + | - | + | - |
| L-Galacturonic Acid Lactone | + | + | - | + | - |
| D-Gluconic Acid | + | + | + | + | + |
| D-Glucuronic Acid | + | + | - | + | - |
| Glucuronamide | + | + | - | + | + |
| Mucic Acid | + | + | + | + | + |
| Quinic Acid | + | + | + | + | - |
| D-Saccharic Acid | + | + | + | + | - |
| *p*-Hydroxy-Phenylacetic Acid | - | - | + | - | - |
| Methyl Pyruvate | + | + | + | + | + |
| D-Lactic Acid Methyl Ester | - | - | - | - | - |
| L-Lactic Acid | + | + | + | + | + |
| Citric Acid | + | + | + | + | + |
| α -Keto-Glutaric Acid | + | + | + | + | + |
| D-Malic Acid | + | + | + | + | + |
| L-Malic Acid | + | + | + | + | + |
| Bromo-Succinic Acid | + | + | + | + | + |
| Tween 40 | + | + | + | + | - |
| Γ-Amino-Butyric Acid | + | + | + | + | + |
| α-Hydroxy-Butyric Acid | + | + | + | + | + |
| β -Hydroxy-D,L-Butyric Acid | + | + | + | + | + |
| α -Keto-Butyric Acid | + | + | - | - | - |
| Acetoacetic Acid | + | + | - | - | - |
| Propionic Acid | + | + | + | + | + |
| Acetic Acid | + | + | + | + | + |
| Formic Acid | + | + | + | + | + |
| **Other Biolog Gen III tests** | **1** | **2** | **3** | **4** | **5** |
| pH 6 | + | + | + | + | + |
| pH 5 | + | + | + | + | + |
| 1% NaCl | + | + | + | + | + |
| 4% NaCl | + | + | - | + | + |
| 8% NaCl | - | - | - | + | - |
| 1% Sodium Lactate | + | + | + | + | + |
| Fusidic Acid | + | + | + | + | + |
| D-Serine | + | + | + | + | + |
| Troleandomycin | + | + | + | + | + |
| Rifamycin Sv | + | + | + | + | + |
| Minocycline | - | - | - | - | - |
| Lincomycin | + | + | + | + | + |
| Guanidine Hcl | + | + | + | + | + |
| Niaproof 4 | + | + | + | + | + |
| Vancomycin | + | + | + | + | + |
| Tetrazolium Violet | + | + | + | + | + |
| Tetrazolium Blue | + | + | + | + | + |
| Nalidixic Acid | + | + | + | + | + |
| Lithium Chloride | + | + | + | + | + |
| Potassium Tellurite | + | + | + | + | + |
| Aztreonam | + | + | + | + | + |
| Sodium Butyrate | + | - | - | - | - |
| Sodium Bromate | + | + | - | + | + |
| **API20 NE feature** | **1** | **2** | **3** | **4** | **5** |
| Nitrate Reduction | - | + | + | + | + |
| Indole Production | - | - | - | - | - |
| Glucose Fermentation | - | - | - | - | - |
| Arginine Dehydrolase | - | - | - | + | - |
| Urease | - | - | - | - | - |
| Esculin Hydrolysis | - | - | - | - | - |
| Gelatin Hydrolysis | - | - | - | - | - |
| β-Galactosidase | - | - | - | - | - |
| Glucose | + | + | + | + | + |
| Arabinose | - | + | + | + | - |
| Mannose | + | + | + | + | - |
| Mannitol | + | - | + | - | + |
| N-Acetyl-Glucosamine | + | + | + | - | + |
| Maltose | - | - | - | - | - |
| Potassium Gluconate | + | + | + | + | + |
| Capric Acid | + | + | + | + | + |
| Adipic Acid | - | - | - | - | - |
| Malate | + | + | + | + | + |
| Trisodium Citrate | + | + | + | + | + |
| Phenylacetic Acid | + | + | + | + | + |
| **Other tests** | **1** | **2** | **3** | **4** | **5** |
| SDS degradation | + | + | - | - | + |
| Fluorescence on King B medium | + | + | + | + | + |
| Oxidase activity | + | + | + | + | + |
| Catalase activity | + | + | + | + | + |

Lanes: 1, *P. laurylsulfatophila* AP3_16^T^; 2, *P. laurylsulfatovorans* AP3_22^T^; 3, *P. jessenii* CIP 105724^T^; 4, *P. umsongensis* DSM 16611^T^; 5, *P. vancouverensis*  DSM 17555^T^. +, positive; -, negative.

**Table S4**. Cellular fatty acid composition (%) derived from FAME analysis of *P. laurylsulfatophila* AP3_16^T^ and the type strains of closely related *Pseudomonas* species. All strains were tested in the same culture conditions described in the Materials and Methods section.

| **Fatty acid** | **1** | **2** | **3** | **4** | **5** | **6** | **7** |
| --- | --- | --- | --- | --- | --- | --- | --- |
| C_10:0 3-OH_ | 4.02 | 3.89 | 3.59 | 4.09 | 4.53 | 5.19 | 5.53 |
| C_12:0_ | 3.17 | 4.09 | 4.52 | 1.19 | 3.45 | 3.60 | 3.41 |
| C_12:0 2-OH_ | 4.07 | 3.17 | 2.68 | 5.51 | 3.97 | 3.38 | 4.13 |
| C_12:0_ _3-OH_ | 4.63 | 4.22 | 3.91 | 4.34 | 4.72 | 6.20 | 5.40 |
| C_12:1_ _3-OH_ | 0.27 | 0.26 | 0.23 | – | 0.28 | 1.86 | 1.22 |
| C_16:0_ | 31.67 | 31.55 | 33.77 | 32.74 | 31.13 | 28.33 | 31.09 |
| C_17:0 cyclo_ | 1.26 | 1.31 | 0.75 | 1.52 | 3.54 | 2.71 | 14.46 |
| C_18:1 ω7c_ | 11.42 | 11.22 | 10.56 | 12.52 | 11.89 | 11.88 | 10.20 |
| Summed feature 2^*^ | 0.14 | 0.13 | 0.09 | – | – | 1.31 | 0.56 |
| Summed feature 3^**^ | 38.13 | 38.81 | 38.59 | 37.05 | 35.32 | 32.63 | 21.82 |

Lanes: 1, *P. laurylsulfatophila* AP3_16^T^; 2, *P. laurylsulfatovorans* AP3_22^T^; 3, *P. jessenii* DSM 17150^T^; 4, *P. baetica* DSM 26532^T^; 5, *P. reinekei* DSM 18361^T^ ; 6, *P. umsongensis* DSM 16611^T^; 7, *P. vancouverensis* DSM 17555^T^.

^*^- contains an unidentified fatty acid with an equivalent chain-length of 10.928 and/or C_12:0 ALDE_

^**^- contains a mixture of C_16:1 ω7c_ and/or C_15:0 iso 2OH_

Only results with amounts higher than 1% for at least one strain are presented.

**Table S5.** Comparison of gene counts associated with the general COGs (Clusters of Orthologous Groups) functional categories for the five compared genomes. Association of genes to COG categories for each genome was done using COGNIZER software.

| **Code** | **Description** | **1** | | **2** | | **3** | | **4** | | **5** | |
| --- | --- | --- | --- | --- | --- | --- | --- | --- | --- | --- | --- |
|  |  | **Value** | **%age** | **Value** | **%age** | **Value** | **%age** | **Value** | **%age** | **Value** | **%age** |
| A | RNA processing and modification | 1 | 0.02 | 1 | 0.02 | 1 | 0.02 | 1 | 0.02 | 1 | 0.02 |
| B | Chromatin structure and dynamics | 8 | 0.13 | 8 | 0.13 | 6 | 0.10 | 4 | 0.07 | 6 | 0.1 |
| C | Energy production and conversion | 633 | 10.42 | 634 | 10.36 | 625 | 10.38 | 686 | 11.8 | 620 | 10.46 |
| D | Cell cycle control, cell division, chromosome partitioning | 60 | 0.99 | 55 | 0.90 | 59 | 0.98 | 49 | 0.84 | 56 | 0.94 |
| E | Amino acid transport and metabolism | 1198 | 19.72 | 1230 | 20.10 | 1175 | 19.52 | 1198 | 20.61 | 1211 | 20.43 |
| F | Nucleotide transport and metabolism | 170 | 2.8 | 181 | 2.96 | 165 | 2.74 | 167 | 2.87 | 185 | 3.12 |
| G | Carbohydrate transport and metabolism | 456 | 7.51 | 510 | 8.34 | 496 | 8.24 | 514 | 8.84 | 439 | 7.4 |
| H | Coenzyme transport and metabolism | 290 | 4.77 | 282 | 4.61 | 280 | 4.65 | 296 | 5.09 | 278 | 4.69 |
| I | Lipid transport and metabolism | 452 | 7.44 | 446 | 7.29 | 426 | 7.08 | 532 | 9.15 | 437 | 7.37 |
| J | Translation, ribosomal structure and biogenesis | 286 | 4.71 | 284 | 4.64 | 277 | 4.60 | 285 | 4.9 | 285 | 4.81 |
| K | Transcription | 800 | 13.17 | 759 | 12.41 | 783 | 13.01 | 749 | 12.88 | 762 | 12.85 |
| L | Replication, recombination and repair | 373 | 6.14 | 380 | 6.21 | 333 | 5.53 | 360 | 6.19 | 329 | 5.55 |
| M | Cell wall/membrane/envelope biogenesis | 545 | 8.97 | 478 | 7.81 | 526 | 8.74 | 460 | 7.91 | 535 | 9.02 |
| N | Cell motility | 207 | 3.41 | 213 | 3.48 | 202 | 3.36 | 215 | 3.7 | 218 | 3.68 |
| O | Posttranslational modification, protein turnover, chaperones | 278 | 4.58 | 284 | 4.64 | 275 | 4.57 | 282 | 4.85 | 279 | 4.71 |
| P | Inorganic ion transport and metabolism | 657 | 10.82 | 674 | 11.02 | 619 | 10.29 | 635 | 10.92 | 684 | 11.54 |
| Q | Secondary metabolites biosynthesis, transport and catabolism | 371 | 6.11 | 397 | 6.49 | 364 | 6.05 | 401 | 6.9 | 337 | 5.68 |
| R | General function prediction only | 1157 | 19.05 | 1199 | 19.60 | 1192 | 19.80 | 1265 | 21.76 | 1172 | 19.77 |
| S | Function unknown | 589 | 9.7 | 630 | 10.30 | 611 | 10.15 | 647 | 11.13 | 589 | 9.93 |
| T | Signal transduction mechanisms | 649 | 10.68 | 629 | 10.28 | 598 | 9.94 | 666 | 11.46 | 650 | 10.96 |
| U | Intracellular trafficking, secretion, and vesicular transport | 197 | 3.24 | 180 | 2.94 | 188 | 3.12 | 195 | 3.35 | 229 | 3.86 |
| V | Defense mechanisms | 164 | 2.7 | 145 | 2.37 | 167 | 2.77 | 142 | 2.44 | 163 | 2.75 |
| - | Not in COG | 1283 | 21.12 | 1324 | 21.64 | 1309 | 21.75 | 1023 | 17.6 | 1199 | 20.22 |

Lanes: 1,*P. laurylsulfatophila* AP3_16^T^; 2, *P. laurylsulfatovorans* AP3_22^T^; 3, *P. jessenii* CIP 105724^T^; 4, *P. umsongensis* DSM 16611^T^; 5, *P. vancouverensis* DSM 17555^T^

**Table S6.** Statistically significant up and down regulated genes during *P. laurylsulfatovorans* AP3_22^T^ exposure to sodium dodecyl sulfate.

| *T5 time point* | | | |
| --- | --- | --- | --- |
| **Locus** | **log_2_FoldChange** | **p_adj_** | **Annotation** |
| B0D71_15235 | 2.226 | 1.57E-09 | polyphosphate kinase 2 |
| B0D71_14720 | 2.171 | 1.29E-07 | hypothetical protein |
| B0D71_19840 | 1.757 | 3.58E-12 | oxidoreductase |
| B0D71_25665 | 1.605 | 1.83E-07 | hypothetical protein |
| B0D71_26625 | 1.492 | 3.48E-10 | amidase |
| B0D71_24675 | 1.451 | 1.05E-09 | iron ABC transporter permease |
| B0D71_08255 | 1.415 | 2.95E-17 | plasmid stabilization protein ParE |
| B0D71_21930 | 1.386 | 4.32E-06 | hypothetical protein |
| B0D71_19065 | 1.302 | 4.32E-06 | uracil permease |
| B0D71_17945 | 1.266 | 8.40E-10 | cardiolipin synthase |
| B0D71_12750 | 1.233 | 3.55E-06 | serine protease |
| B0D71_19845 | 1.182 | 1.85E-05 | glycerol-3-phosphate dehydrogenase |
| B0D71_16700 | 1.018 | 5.07E-06 | thymidylate synthase |
| B0D71_10800 | 1.006 | 8.47E-05 | hypothetical protein |
| B0D71_22785 | -1.044 | 8.40E-06 | acyl-CoA desaturase |
| B0D71_08785 | -1.151 | 6.31E-08 | LysR family transcriptional regulator |
| B0D71_16090 | -1.407 | 7.31E-05 | hypothetical protein |
| *T30 time point* | | | |
| **Locus** | **log_2_FoldChange** | **p_adj_** | **Annotation** |
| B0D71_26505 | 4.848 | 1.54E-19 | NUDIX hydrolase |
| B0D71_06340 | 2.751 | 6.53E-05 | phosphatidic acid phosphatase |
| B0D71_01775 | 2.112 | 8.07E-05 | lactoylglutathione lyase |
| B0D71_25530 | 1.743 | 1.61E-06 | poly(3-hydroxyalkanoate) granule-associated protein PhaF |
| B0D71_17685 | 1.490 | 8.58E-05 | phosphate-binding protein |
| B0D71_29615 | 1.386 | 4.49E-08 | LysR family transcriptional regulator |
| B0D71_21830 | 1.327 | 5.57E-06 | transcriptional regulator |
| B0D71_24460 | 1.283 | 7.64E-15 | hypothetical protein |
| B0D71_14915 | 1.271 | 1.02E-06 | hypothetical protein |
| B0D71_21570 | 1.125 | 9.80E-06 | anhydro-N-acetylmuramic acid kinase |
| B0D71_20510 | 1.115 | 2.79E-08 | FAD-dependent oxidoreductase |
| B0D71_18875 | 1.050 | 4.72E-05 | hypothetical protein |
| B0D71_21800 | 1.041 | 6.28E-05 | 30S ribosomal protein S21 |
| B0D71_10760 | -1.335 | 4.60E-15 | 2-oxoglutarate dehydrogenase E1 component |
| B0D71_14525 | -1.361 | 2.58E-16 | AraC family transcriptional regulator |
| B0D71_21080 | -1.443 | 1.58E-05 | pseudouridine synthase |
| B0D71_14675 | -1.495 | 2.49E-07 | hypothetical protein |
| B0D71_16870 | -1.499 | 1.11E-11 | hypothetical protein |
| B0D71_21645 | -1.574 | 2.47E-07 | aminodeoxychorismate/anthranilate synthase component II |
| B0D71_22785 | -1.807 | 3.27E-16 | acyl-CoA desaturase |
| B0D71_12785 | -1.810 | 3.72E-08 | carbon storage regulator |
| B0D71_10145 | -1.833 | 4.21E-07 | LTXXQ domain protein |
| B0D71_08785 | -1.854 | 8.12E-35 | LysR family transcriptional regulator |
| B0D71_28295 | -2.078 | 1.39E-21 | flagellar biosynthesis anti-sigma factor FlgM |
| B0D71_10300 | -2.088 | 1.33E-08 | branched-chain alpha-keto acid dehydrogenase subunit E2 |
| B0D71_08420 | -2.374 | 5.77E-05 | 2-methylisocitrate lyase |
| B0D71_10120 | -2.840 | 3.96E-45 | hypothetical protein |
| *T60 time point* |  |  |  |
| **Locus** | **log_2_FoldChange** | **p_adj_** | **Annotation** |
| B0D71_10120 | 2.772 | 2.06E-51 | hypothetical protein |
| B0D71_04505 | 2.466 | 2.69E-11 | DUF1289 domain-containing protein |
| B0D71_10315 | 2.314 | 2.92E-05 | flagellar export chaperone FliS |
| B0D71_21540 | 2.141 | 2.69E-11 | NAD-dependent dehydratase |
| B0D71_10300 | 2.074 | 1.56E-06 | branched-chain alpha-keto acid dehydrogenase subunit E2 |
| B0D71_21630 | 2.044 | 5.38E-05 | lipoate--protein ligase |
| B0D71_10345 | 1.854 | 1.13E-06 | flagellar M-ring protein FliF |
| B0D71_21850 | 1.809 | 2.37E-06 | amine oxidase |
| B0D71_30275 | 1.639 | 2.29E-06 | methyl-accepting chemotaxis protein |
| B0D71_29195 | 1.570 | 3.74E-05 | type II secretion system protein |
| B0D71_17780 | 1.539 | 1.13E-06 | GNAT family N-acetyltransferase |
| B0D71_10130 | 1.497 | 3.67E-08 | potassium transporter TrkH |
| B0D71_13645 | 1.467 | 6.05E-08 | hypothetical protein |
| B0D71_25805 | 1.447 | 5.76E-14 | cobalamin biosynthesis protein CobQ |
| B0D71_23825 | 1.410 | 4.22E-06 | methyl-accepting chemotaxis protein |
| B0D71_24910 | 1.347 | 4.77E-05 | DNA-binding protein |
| B0D71_07260 | 1.342 | 4.42E-06 | hypothetical protein |
| B0D71_08785 | 1.284 | 4.11E-22 | LysR family transcriptional regulator |
| B0D71_14675 | 1.187 | 4.17E-05 | hypothetical protein |
| B0D71_16870 | 1.178 | 2.86E-08 | hypothetical protein |
| B0D71_20535 | 1.018 | 1.54E-06 | ATP-dependent chaperone ClpB |
| B0D71_28350 | 1.014 | 4.58E-07 | hypothetical protein |
| B0D71_17085 | 1.009 | 7.74E-05 | glycerol-3-phosphate transporter |
| B0D71_12865 | -1.027 | 3.74E-05 | DNA-binding protein |
| B0D71_24425 | -1.193 | 2.37E-06 | 50S ribosomal protein L32 |
| B0D71_06620 | -1.204 | 7.24E-08 | 30S ribosomal protein S15 |
| B0D71_29615 | -1.274 | 7.06E-06 | LysR family transcriptional regulator |
| B0D71_24460 | -1.316 | 4.08E-09 | hypothetical protein |
| B0D71_19845 | -1.378 | 1.49E-05 | glycerol-3-phosphate dehydrogenase |
| B0D71_24405 | -1.431 | 2.56E-05 | acyl carrier protein |
| B0D71_19855 | -1.525 | 1.39E-05 | glycerol kinase |
| B0D71_19840 | -1.707 | 4.08E-09 | oxidoreductase |
| B0D71_25530 | -1.708 | 3.65E-07 | poly(3-hydroxyalkanoate) granule-associated protein PhaF |
| B0D71_18330 | -1.965 | 4.26E-06 | 50S ribosomal protein L34 |
| B0D71_21800 | -1.982 | 4.17E-05 | 30S ribosomal protein S21 |
| B0D71_26505 | -3.535 | 1.35E-07 | NUDIX hydrolase |
| *T150 time point* | | | |
| **Locus** | **log_2_FoldChange** | **p_adj_** | **Annotation** |
| B0D71_09765 | 2.713 | 2.69E-11 | hypothetical protein |
| B0D71_17480 | 2.630 | 2.73E-09 | 50S ribosomal protein L28 |
| B0D71_25585 | 2.562 | 2.99E-05 | 50S ribosomal protein L31 |
| B0D71_08225 | 2.542 | 1.78E-05 | amino acid ABC transporter ATP-binding protein |
| B0D71_12030 | 2.512 | 3.59E-07 | hypothetical protein |
| B0D71_17475 | 2.500 | 5.21E-06 | 50S ribosomal protein L33 |
| B0D71_23925 | 2.439 | 4.04E-06 | chain-length determining protein |
| B0D71_08560 | 2.392 | 8.40E-08 | 30S ribosomal protein S2 |
| B0D71_23595 | 2.390 | 5.69E-05 | dioxygenase |
| B0D71_29915 | 2.387 | 8.39E-08 | 50S ribosomal protein L10 |
| B0D71_06850 | 2.273 | 6.37E-05 | malate transporter |
| B0D71_21800 | 2.177 | 1.53E-06 | 30S ribosomal protein S21 |
| B0D71_11870 | 2.081 | 4.36E-07 | amino acid ABC transporter substrate-binding protein |
| B0D71_20005 | 1.949 | 7.89E-09 | co-chaperone GroES |
| B0D71_24040 | 1.949 | 7.82E-10 | 30S ribosomal protein S1 |
| B0D71_24405 | 1.937 | 1.49E-05 | acyl carrier protein |
| B0D71_12850 | 1.909 | 7.67E-05 | hypothetical protein |
| B0D71_29795 | 1.900 | 1.22E-07 | 50S ribosomal protein L6 |
| B0D71_06620 | 1.860 | 1.62E-05 | 30S ribosomal protein S15 |
| B0D71_06495 | 1.773 | 7.82E-10 | hypothetical protein |
| B0D71_23665 | 1.772 | 6.44E-06 | elongation factor P |
| B0D71_12805 | 1.766 | 7.41E-06 | L-asparaginase |
| B0D71_24045 | 1.674 | 4.21E-06 | cytidylate kinase |
| B0D71_20665 | 1.671 | 7.21E-05 | 50S ribosomal protein L27 |
| B0D71_29775 | 1.592 | 1.89E-05 | 50S ribosomal protein L15 |
| B0D71_28155 | 1.580 | 4.31E-05 | hypothetical protein |
| B0D71_21810 | 1.570 | 2.21E-08 | hypothetical protein |
| B0D71_10120 | 1.557 | 2.36E-05 | hypothetical protein |
| B0D71_12865 | 1.547 | 5.63E-06 | DNA-binding protein |
| B0D71_27080 | 1.543 | 4.31E-05 | cold-shock protein |
| B0D71_14720 | 1.518 | 2.80E-06 | hypothetical protein |
| B0D71_08240 | 1.515 | 5.69E-05 | amino acid ABC transporter substrate-binding protein |
| B0D71_24460 | 1.487 | 6.51E-05 | hypothetical protein |
| B0D71_25805 | 1.408 | 4.23E-05 | cobalamin biosynthesis protein CobQ |
| B0D71_17325 | 1.306 | 5.92E-05 | hypothetical protein |
| B0D71_18785 | 1.300 | 7.69E-06 | lysine methyltransferase |
| B0D71_06515 | 1.281 | 1.78E-05 | molecular chaperone DnaK |
| B0D71_17465 | -1.490 | 4.31E-05 | aldehyde dehydrogenase PuuC |
| B0D71_23520 | -1.534 | 4.31E-05 | urocanate hydratase |
| B0D71_23545 | -1.596 | 5.69E-05 | histidine ammonia-lyase |
| B0D71_02025 | -1.619 | 6.38E-06 | acyl-CoA dehydrogenase |
| B0D71_27215 | -1.634 | 3.12E-05 | sugar ABC transporter substrate-binding protein |
| B0D71_09080 | -1.678 | 3.59E-07 | leucine ABC transporter subunit substrate-binding protein LivK |
| B0D71_04765 | -1.703 | 3.59E-07 | dihydrolipoyl dehydrogenase |
| B0D71_14695 | -1.707 | 1.36E-07 | 3-methylcrotonyl-CoA carboxylase |
| B0D71_14690 | -1.838 | 5.64E-10 | Cro/Cl family transcriptional regulator |
| B0D71_24790 | -1.842 | 4.80E-07 | branched chain amino acid ABC transporter substrate-binding protein |
| B0D71_27235 | -1.878 | 4.21E-06 | porin |
| B0D71_04755 | -1.886 | 5.05E-07 | alpha-ketoacid dehydrogenase subunit beta |
| B0D71_17200 | -1.916 | 2.36E-06 | TIGR02444 family protein |
| B0D71_04760 | -1.924 | 3.22E-07 | branched-chain alpha-keto acid dehydrogenase subunit E2 |
| B0D71_26535 | -1.966 | 7.82E-10 | hypothetical protein |
| B0D71_12315 | -2.036 | 2.36E-07 | hypothetical protein |
| B0D71_04750 | -2.073 | 3.81E-08 | 3-methyl-2-oxobutanoate dehydrogenase (2-methylpropanoyl-transferring) subunit alpha |
| B0D71_02005 | -2.075 | 4.82E-06 | 3-hydroxyacyl-CoA dehydrogenase |
| B0D71_14705 | -2.092 | 2.37E-08 | methylcrotonoyl-CoA carboxylase |
| B0D71_26630 | -2.171 | 2.36E-07 | bile acid:sodium symporter |
| B0D71_10220 | -2.217 | 1.75E-05 | 4a-hydroxytetrahydrobiopterin dehydratase |
| B0D71_16690 | -2.268 | 6.40E-09 | heavy metal translocating P-type ATPase |
| B0D71_25390 | -2.284 | 6.63E-05 | ABC transporter permease |
| B0D71_30115 | -2.326 | 1.82E-08 | methylmalonate-semialdehyde dehydrogenase (CoA acylating) |
| B0D71_01810 | -2.340 | 3.22E-07 | chemotaxis protein |
| B0D71_15890 | -2.394 | 1.06E-07 | acetyl-CoA acetyltransferase |
| B0D71_02030 | -2.435 | 1.97E-05 | crotonase |
| B0D71_03255 | -2.526 | 1.80E-07 | sensor histidine kinase |
| B0D71_06230 | -2.528 | 1.57E-13 | methylmalonate-semialdehyde dehydrogenase (CoA acylating) |
| B0D71_21315 | -2.535 | 7.41E-06 | aldehyde dehydrogenase |
| B0D71_30120 | -2.547 | 3.09E-09 | AMP-dependent synthetase |
| B0D71_07230 | -2.571 | 7.36E-08 | fumarylacetoacetase |
| B0D71_20835 | -2.607 | 9.29E-07 | MFS transporter |
| B0D71_07225 | -2.622 | 1.82E-09 | homogentisate 1.2-dioxygenase |
| B0D71_05950 | -2.655 | 7.90E-09 | glutamine synthetase |
| B0D71_27095 | -2.689 | 2.61E-13 | glycine dehydrogenase (aminomethyl-transferring) |
| B0D71_07240 | -2.750 | 3.03E-17 | hypothetical protein |
| B0D71_19620 | -2.762 | 8.46E-07 | hypothetical protein |
| B0D71_02010 | -2.839 | 1.08E-06 | acetyl-CoA acetyltransferase |
| B0D71_30110 | -3.094 | 3.59E-05 | 3-hydroxyisobutyrate dehydrogenase |
| B0D71_05945 | -3.273 | 1.68E-07 | aldehyde dehydrogenase PuuC |
| B0D71_21685 | -3.330 | 6.81E-13 | spermidine/putrescine ABC transporter substrate-binding protein |
| B0D71_06235 | -3.375 | 2.62E-13 | 3-hydroxyisobutyrate dehydrogenase |
| B0D71_27815 | -4.399 | 4.05E-16 | magnesium-translocating P-type ATPase |
